# Morpho-molecular and nutritional profiling for yield improvement and value addition of indigenous aromatic *Joha* rice of Assam

**DOI:** 10.1038/s41598-023-42874-9

**Published:** 2024-02-12

**Authors:** Dibosh Bordoloi, Debojit Sarma, Nagendra Sarma Barua, Ranjan Das, Bikram Kishore Das

**Affiliations:** 1 AAU-Zonal Research Station, Assam Agricultural University, Karimganj, 788712 India; 2 Department of Plant Breeding and Genetics, Assam Agricultural University, Jorhat, 785013 India; 3 Department of Crop Physiology, Assam Agricultural University, Jorhat, 785013 India; 4 Nuclear Agriculture and Biotechnology Division, Bhabha Atomic Research Centre, Mumbai, 400085 India

**Keywords:** Genetics, Molecular biology, Plant sciences

## Abstract

Short-grain aromatic *Joha* rice of Assam is a unique class of specialty rice having tremendous potential in domestic and international markets. The poor yielding ability of Assam's *Joha* rice demands its systematic characterization for an effective breeding program. This study investigates the morphological, molecular and biochemical profiles of twenty popular *Joha* (aromatic) rice cultivars indigenous to Assam. Distinctiveness, Uniformity and Stability (DUS) characterization of the cultivars revealed polymorphism in thirty-seven traits, establishing distinctiveness for their utilization in breeding programs. Unweighted Neighbor Joining (UNJ) clustering based on usual Euclidean distances for the polymorphic morphological markers grouped the cultivars into three clusters with eight, eleven, and one genotypes. The *Joha* rice cultivars showed significant differences for all the quantitative traits except for panicle length. The genotypic and phenotypic coefficients of variability (GCV & PCV) were high for grain yield ha^−1^ (24.62 & 24.85%) and filled grains panicle^−1^ (23.69 & 25.02%). Mahalanobis D^2^ analysis revealed three multi-genotypic and four mono-genotypic clusters of the cultivars. The first five principal components explain 85.87% of the variation among the cultivars for the traits under study; filled grain panicle^−1^ (0.91) and stem thickness (0.55) positively contributed to the first PC. The cultivars' average polyunsaturated fatty acids were 37.9% oleic acid, 39.22% linoleic acid, and 0.5% linolenic acid. *Kon Joha* *4* and *Ronga Joha* contained the highest iron (82.88 mg kg^−1^) and zinc (47.39 mg kg^−1^), respectively. *Kalijeera*, *Kunkuni Joha*, *Kon Joha-5*, *Manimuni Joha* and *Kon Joha-2* accorded a strong aroma. PCR amplified 174 alleles with a mean value 2.64 across the 66 polymorphic SSR markers. PIC values ranged from 0.091 to 0.698, with an average of 0.326. The highly informative (PIC > 0.50) markers were RM316, RM283, RM585, RM1388, RM3562, RM171, R1M30, RM118, RM11and RM29 for identification of the twenty aromatic rice cultivars. PCR amplification of 27 SSR markers identified 28 unique alleles (97–362 bp) in 13 *Joha* rice cultivars, which can help their identification/DNA fingerprinting. The UNJ clustering based on Jaccard's coefficients classified the cultivars into three distinct clusters with eight, ten, and two genotypes. Our study revealed the nutritional richness of these specialty *Joha* rice cultivars and sufficient scope for yield enhancement through their interbreeding to keep quality intact.

## Introduction

Globally, rice is planted in approximately 162 million ha and 755 million tons of produce are harvested annually^1^. Ninety per cent of the production and consumption of the world's rice occur in Asia. India has the world's largest area under rice with 44.0 million ha and is the second-largest producer (96.0 million tonnes in 2010), next only to China, contributing 21.5% of global rice production. The FAO^2^ estimated a 70 per cent more food requirement for over nine billion people expected to inhabit planet Earth by 2050. As the staple food for most Indians, rice's future demand will increase with the growing population, projected at 1.378 billion by 2030^3^. In Assam, rice occupies approximately 70% of the total cropped area, dominating the state's agriculture^4^. Rice, the primary agricultural GDP source, plays a significant role in the state economy.

Aromatic rice is a significantly small but important rice subgroup that has gained popularity among consumers. The fragrance of scented rice is a crucial feature that increases its popularity in the international market^5^. Aromatic rice is considered the best quality and fetches a much higher price than non-aromatic rice^6^. A unique class of fragrant rice grown as winter rice in Assam is very popular and highly valued due to its quality, known as *Joha* rice in Assam. *Joha* rice possesses a superfine kernel, unique aroma, better cooking properties, and excellent palatability^7^. The aromatic accession**s** from Assam, Manipur and Sikkim belong to the *Indica* group^8^. The aroma (fragrance) of *Joha* rice cultivars is due to the presence of a non-functional betaine aldehyde dehydrogenase 2 (BADH2), which also lowers grain yield^9^.

*Joha* rice is generally a poor yielder, late maturing, tall, and susceptible to lodging. It occupies approximately 5 per cent of the *Sali* rice area, with an average yield of 1–1.5 per ha^7^. Modern high-yielding varieties have replaced the *Joha* rice cultivars, depleting this valuable genetic wealth of aromatic and fine-rice genotypes. However, its aroma and unique cooking quality allowed a global market for *Joha* rice to enter the European market in 2007.

Furthermore, Assam's non-basmati *Joha* rice cultivars were granted Geographical Indication (GI) tags in 2017 (http://ipindiaservices.gov.in). Information on genetic variability encompassing morphological, biochemical and molecular characterization of Assam's *Joha* rice is meagre if present. Genetic diversity studies among the aromatic germplasm of Assam would help their improvement through crossbreeding and identification of pre-breeding materials^10^ and would preserve information on this unique rice class for the future.

Agro-morphological characterization of rice germplasm is fundamental to providing information for plant breeding programmes^11^. The nutritional status of rice is becoming increasingly important among consumers because of deficiency disorders, necessitating more knowledge regarding the nutritional composition of different rice varieties in Assam. However, the characterization of rice cultivars in terms of composition and physicochemical aspects is relatively scarce. Rice is a leading food crop with a different nutritional status that helps alleviate poverty^12^. Proximate analysis is an essential parameter for the routine description of foodstuffs. This analysis comprises six fractions: amylose, crude protein, iron, zinc, and fatty acid. The proximate composition, sensory, gelatinization temperature, and alkali digestion analysis of rice help identify nutritionally complete rice varieties^13^.

Rice showed a genetically greater extent of diversity. DNA-based molecular markers are essential in assessing genetic variation and elucidating gene relationships between species. Molecular markers have been extensively used in crop improvement research throughout the globe as an appropriate and effective tool for addressing biological parameters in agricultural productivity^14^. Among PCR-based markers, SSR or microsatellite markers are excellent due to their locus of identity, high polymorphism, and multiallelic nature. Moreover, SSR markers are tandemly interspersed repeats throughout the genome that are amplified through primers that flank these regions. Studies to characterize and improve the aromatic rice of Assam are limited, even though this region stores enormous rice diversity. The present study characterized a panel of popular *Joha* rice cultivars grown in Assam's Brahmaputra and Barak valleys using morphological, biochemical, and molecular markers for their usage in yield enhancement breeding. Most cultivars are popular among the farmers of the Brahmaputra Valley, except *Kalijeera* and *Harinarayan*, which are well-liked in the Barak Valley of Assam. Farmers and consumers have particular preferences for these cultivars because of their unique quality; some are believed to have medicinal properties. Barak Valley farmers consume *Kalijeera* rice to improve health, increase appetite, and reduce hearing problems. The inherently poor yield of *Joha* rice is quite discouraging for a high return, even if it fetches a high market price, due to rapidly increasing production costs. Many such known cultivars are already out of cultivation in the farmers' field. This situation demands breeders' immediate attention to mainstream these valuable *Joha* rice cultivars through intensive research for value-addition and yields to sustain their cultivation (Table 1).

**Table 1:** List of indigenous *Joha* rice cultivars used in the investigation.

S. no.	Cultivar name	Pedigree	Origin
1	*Joha (Bihpuria)*	Landrace	Assam
2	*Kalijeera*	Landrace	Assam
3	*Ronga Joha*	Landrace	Assam
4	*Joha (Golaghat)*	Landrace	Assam
5	*Manimuni Joha*	Landrace	Assam
6	*Kon Joha (Moran)*	Landrace	Assam
7	*Keteki Joha*	Savitri/Badshabhog	Assam
8	*Kon Joha-1*	Landrace	Assam
9	*Soru Joha (Tinsukia)*	Landrace	Assam
10	*Kon Joha-2*	Landrace	Assam
11	*Jeera Joha*	Landrace	Assam
12	*Kon Joha-3*	Landrace	Assam
13	*Kon Joha-4*	Landrace	Assam
14	*Kunkuni Joha*	Landrace	Assam
15	*Kon Joha-5*	Landrace	Assam
16	*Local Joha*	Landrace	Assam
17	*Harinarayan*	Landrace	Assam
18	*Kon Joha (Teok)*	Landrace	Assam
19	*Kola Joha*	Landrace	Assam
20	*Kon Joha (Bongaigaon)*	Landrace	Assam

## Results

### Morphological characterization of the cultivars

**Figure 1:**
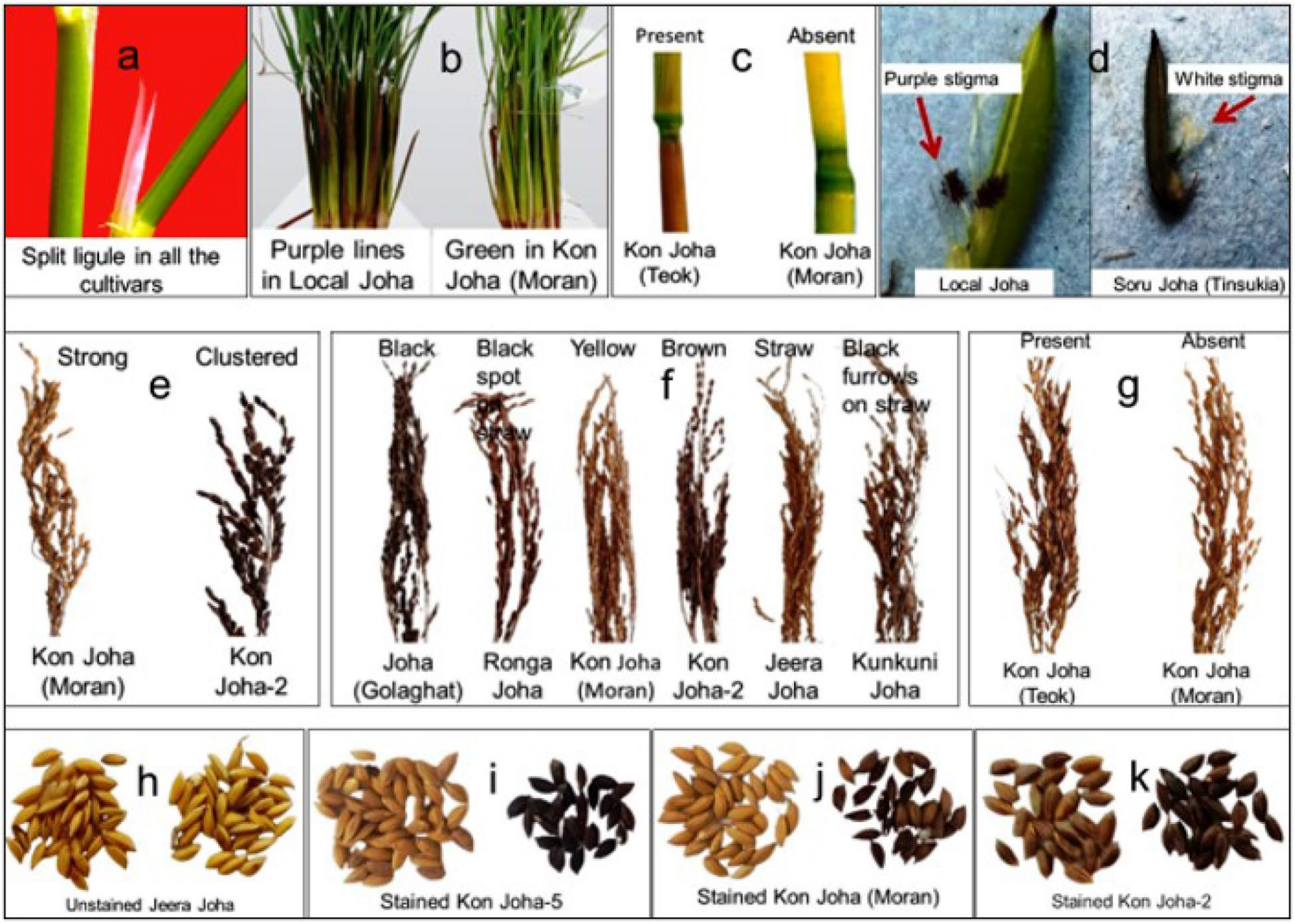
Variations in some morphological characteristics of the *Joha* rice cultivars: (**a**) ligule shape—split in all cultivars; (**b**) basal leaf sheath colour; (**c**) internode colour; (**d**) stigma colour; (**e**) panicle secondary branching; (**f**) lemma palea colour; (**g**) panicle awns; (**h–k**) phenol reaction of the grains.

A total of sixty-two morphological traits characterized the distinctiveness of the twenty *Joha* rice cultivars. Among the characteristics, the spectrum of variability in the twenty cultivars revealed twenty-two monomorphic (Supplementary Table S2) and thirty-seven polymorphic markers (Table 2; Fig. 1).

**Table 2:** Distribution of the *Joha* rice cultivars based on polymorphic characteristics.

S. no.	Characteristics	States	Note	%
1	Basal leaf: sheath colour	Green	1	95
		Purple lines	3	5
2	Leaf: distribution of anthocyanin colouration	On margins only	1	40
		On tips only	2	60
3	Leaf pubescence of blade surface	Weak	3	85
		Medium	5	15
4	Leaf: length of blade	Medium	5	25
		Long	7	75
5	Flag leaf: attitude of blade (early observation)	Semi-erect	3	85
		Horizontal	5	15
6	Spikelet: density of pubescence of lemma	Medium	5	85
		Strong	7	15
7	Lemma: anthocyanin colouration of keel	Absent	1	30
		Strong	7	20
		Very strong	9	50
8	Lemma: anthocyanin colouration of area below apex	Absent	1	30
		Strong	7	20
		Very strong	9	50
9	Lemma: anthocyanin colouration of apex	Absent	1	30
		Medium	5	5
		Strong	7	25
		Very strong	9	40
10	Spikelet: colour of stigma	White	1	80
		Light green	2	10
		Yellow	3	5
		Purple	5	5
11	Stem thickness	Thin	3	50
		Medium	5	50
12	Stem: length (excluding panicle; excluding floating rice)	Very short	1	10
		Short	3	90
13	Stem: anthocyanin colouration of nodes	Absent	1	90
		Present	9	10
14	Stem: intensity of anthocyanin colouration of nodes	Weak	3	5
		Medium	5	5
15	Stem: anthocyanin colouration of internodes	Absent	1	90
		Present	9	10
16	Panicle: length of main axis	Medium	5	40
		Long	7	55
		Very long	9	5
17	Flag leaf: attitude of blade (late observation)	Semi-erect	3	40
		Horizontal	5	60
18	Panicle: number per plant	Few	3	65
		Medium	5	35
19	Spikelet: colour of tip of lemma	Yellowish	2	25
		Brown	3	15
		Black	6	60
20	Lemma and Palea: colour	Straw	1	30
		Brown spot on straw	3	10
		Brown furrows on straw	4	10
		Brown (tawny)	5	5
		Black	9	45
21	Panicle : awns	Absent	1	70
		Present	9	30
22	Panicle: colour of awns (late observation)	Yellowish white	1	10
		Brown	3	5
		Purple	8	5
		Black	9	10
23	Panicle: length of longest awn	Very short	1	10
		Short	3	10
		Very long	9	10
24	Panicle: distribution of awns	Tip only	1	5
		Upper half only	3	10
		Whole length	5	15
25	Panicle: secondary branching	Strong	2	80
		Clustered	3	20
26	Panicle attitude of branches	Semi-erect	5	75
		Semi-erect to spreading	7	25
27	Time maturity (days)	Late	7	85
		Very late	9	15
28	Leaf senescence	Early	3	20
		Medium	5	15
		late	7	65
29	Sterile lemma: colour	Straw	1	45
		Purple	4	55
30	Grain weight of 1000 fully developed grains	Very low	1	25
		Low	3	55
		Medium	5	15
		High	7	5
31	Grain: length	Short	3	70
		Medium	5	30
32	Grain: width	Narrow	3	70
		Medium	5	30
33	Grain: Phenol reaction of lemma	Absent	1	80
		Present	9	20
34	Decorticated grain: length	Short	1	70
		Medium	3	30
35	Decorticated grain: width	Narrow	3	15
		Medium	5	85
36	Decorticated grain: shape (in lateral view)	Short bold	2	65
		Medium slender	3	15
		Long bold	4	15
		Long Slender	5	5
37	Endosperm content of amylose	Low	3	50
		Medium	5	50

### Cluster analysis of the cultivars based on polymorphic DUS characteristics

**Figure 2:**
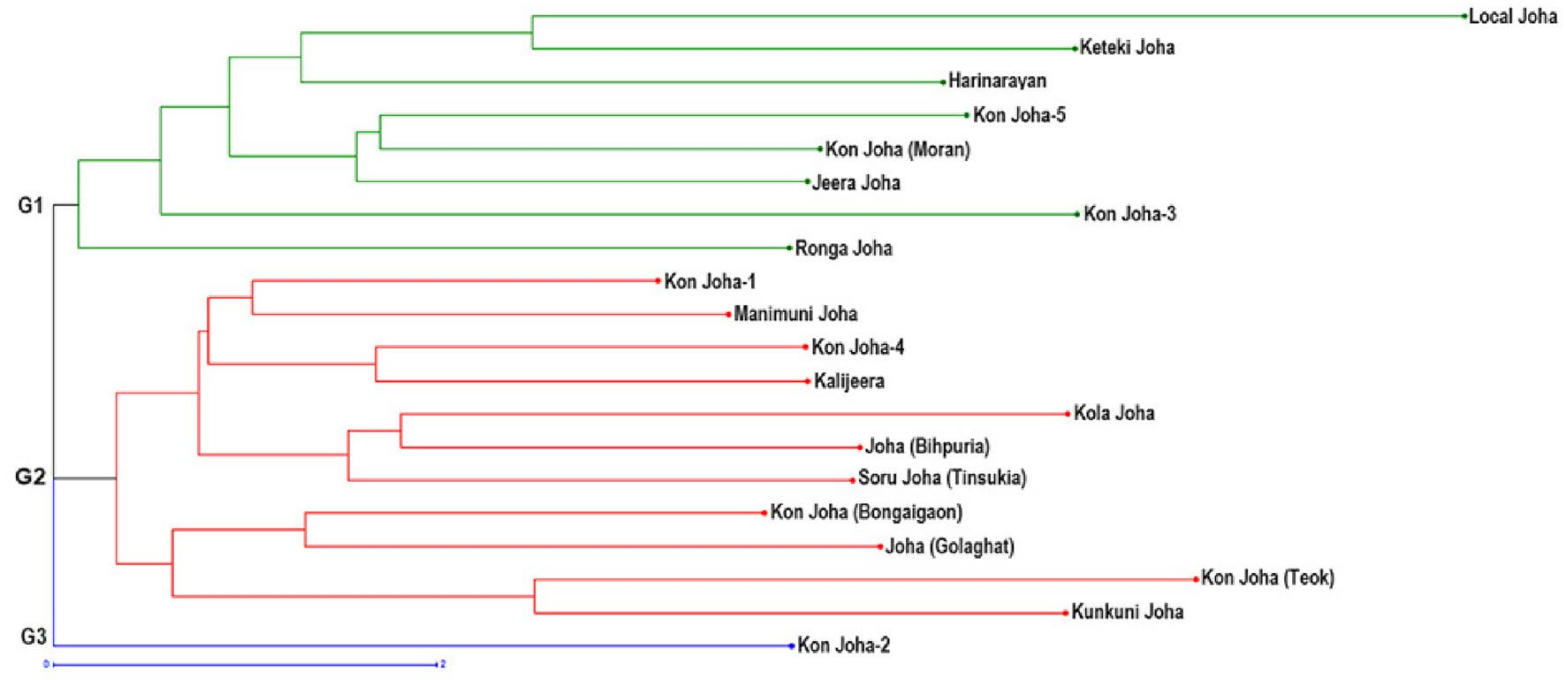
Hierarchical horizontal clustering of the 20 *Joha* rice cultivars using Unweighted Neighbour-Joining (UNJ) method based on usual Euclidean distances estimated from 37 polymorphic morphological markers.

Clustering by unweighted neighbour-joining (UNJ) of the usual Euclidean distance matrix (Supplementary Table S3) based on thirty-seven polymorphic traits grouped the twenty indigenous aromatic rice cultivars into two multi-genotypic clusters and one mono-genotypic cluster (Fig. 2); the clusters were further subdivided into sub clusters with an unequal cultivar distribution. The eight cultivars, viz., *Local Joha*, *Keteki Joha*, *Harinarayan*, *Kon Joha-5*, *Kon Joha (Moran)*, *Jeera Joha*, *Kon Joha-3*, and *Ronga Joha, belonged to G1.* At the same time, *G2* had eleven cultivars viz., *Kon Joha-1*, *Manimuni Joha*, *Kon Joha-4,* *Kalijeera, Kola Joha*, *Joha (Bihpuria)*, *Soru Joha (Tinsukia)*, *Kon Joha (Bongaigaon)*, *Joha (Golaghat)*, *Kon Joha (Teok)* and *Kunkuni Joha, whereas G3* had *Kon Joha-2* alone*.*

### Pooled analysis of variance

The pooled ANOVA over the two years (Supplementary Table S4) revealed that the mean squares due to years were significant for fifteen traits, viz., days to first flowering, flag leaf length, flag leaf breadth, flag leaf area, days to 50% flowering, days to maturity, plant height, productive tillers plant^−1^, filled grains panicle^−1^, spikelet fertility, 1000-grain weights, biological yield plant^−1^, grain yield plant^−1^, harvest index and grain yield kg ha^−1^. The cultivar differences for all the traits except for panicle length were also evident from highly significant mean squares. The year x cultivar interaction component was substantial for days to first flowering, days to 50% flowering, days to maturity, filled grain panicle^−1^, and spikelet fertility.

### Mean performances of the cultivars

The cultivars' mean performances (Supplementary Table S5) identified 11 top-ranking cultivars for the observed traits (Table 3). The cultivars were *Kola Joha* for early flowering/maturing, large grains and high biological yield; *Soru Joha (Tinsukia)* for broad flag leaf, long decorticated grains, wide decorticated grain length/breadth ratio and increased grain yield ha^−1^; *Ronga Joha* for high flag leaf area and high grain yield plant^−1^; *Keteki Joha* for reduced height and high productive tillers; *Kon Joha-5* for high filled grains on panicles and broad grains; *Kon Joha-2* for thick stem wide decorticated grains; *Kon Joha (Teok)* for long flag leaf; *Jeera Joha* for high spikelet fertility; *Kon Joha-3* for long grains; *Local Joha* for wide grain length/breadth ratio; and *Joha (Bihpuria)* for high harvest index. All the *Joha* rice cultivars are typically low to medium tillering and tall.

**Table 3:** Top ranking cultivars with desirable characteristics.

Cultivar	Desirable characteristics
*Kola Joha*	Days to 1st flowering*, days to 50% flowering*, days to maturity*, 1000-grain weights (g), biological yield (g Plant^−1^)
*Soru Joha (Tinsukia)*	Flag leaf breath (cm), decorticated grain length (mm), decorticated grain length/breadth ratio, grain yield (kg ha^−1^)
*Ronga Joha*	Flag leaf area (cm^2^), grain yield (g Plant^−1^)
*Keteki Joha*	Plant height (cm)**, productive tillers Plant^−1^
*Kon Joha-5*	Filled grains Panicle^−1^, grain breadth (mm)
*Kon Joha-2*	Stem thickness (mm), decorticated grain breadth (mm)
*Kon Joha (Teok)*	Flag leaf length (cm)
*Jeera Joha*	Spikelet fertility (%)
*Kon Joha-3*	Grain length (mm)
*Local Joha*	Grain length/breadth ratio
*Joha (Bihpuria)*	Harvest index (%)

*Earliness is desirable.

**Reduced height is desirable to prevent lodging.

### Genetic variability parameters

Grain yield (kg ha^−1^) registered the highest range of variation, followed by flag leaf length, filled grains panicle^−1^, 1000-grain weights, grain length/breadth ratio, grain yield plant^−1^, productive tiller plant^−1^, decorticated grain length, stem thickness, and biological yield plant^−1^ (Table 4). The magnitude of the genotypic and phenotypic coefficients of variability (Table 4) was high (> 20%) for grain yield kg ha^−1^and filled grains panicle^−1^. The variability estimates were moderate (10–20%) for decorticated grain length/breadth ratio,1000-grain weights, grain length/breadth ratio, decorticated grain length, grain length, productive tillers plant^−1^, flag leaf area and stem thickness. The heritability estimates ranged from 37.36 per cent for grain yield plant^−1^ to 99.99 per cent for flag leaf length (Table 4). Most traits exhibited high heritability values (82.64 to 99.99%); the exceptions were productive tillers, harvest index and biological yield with average estimates (> 40%), and grain yield plant^−1^ was low heritable. As a per cent of the mean, the genetic advances were high (> 20%) for grain yield ha^−1^, filled grain panicle^−1^, decorticated grain length/breadth ratio, 1000-grain weights, grain length/breadth ratio, decorticated grain length, grain length, flag leaf area, stem thickness and productive tillers plant^−1^ (Table 4).

**Table 4:** Genetic variability parameters for the traits of the twenty indigenous *Joha* rice cultivars of Assam evaluated during *Sali* season of 2018 and 2019.

Trait	Range	Mean ± SE	GCV (%)	PCV (%)	h^2^_bs_ (%)	GA (5%), As % of mean
Stem thickness (mm)	3.00–4.96	4.09 ± 0.07	10.23	10.37	97.34	20.79
Days to 1st flowering	108.00–123.33	114.51 ± 0.52	3.83	3.86	98.62	7.84
Flag leaf length (cm)	40.84–54.82	47.96 ± 0.05	9.08	9.08	99.99	18.71
Flag leaf breath (cm)	0.75–0.84	0.79 ± 0.01	3.61	3.73	93.80	7.21
Flag leaf area (cm^2^)	24.21–34.0	28.29 ± 0.26	10.62	10.66	99.23	21.79
Days to 50% flowering	112.50–128.50	119.80 ± 0.56	3.93	3.96	98.59	8.05
Days to maturity	144.50–162.03	152.16 ± 0.69	3.35	3.38	98.18	6.85
Plant height (cm)	98.90–142.90	127.80 ± 3.74	7.83	8.36	87.75	15.11
Productive tillers Plant^−1^	7.80–13.80	10.42 ± 1.17	13.15	17.27	58.00	20.64
Filled grains Panicle^−1^	101.73–252.07	177.21 ± 14.24	23.69	25.02	89.69	46.22
Spikelet fertility (%)	78.97–93.03	87.04 ± 1.88	4.72	5.19	82.64	8.83
1000-grain weights (g)	13.02–26.69	17.60 ± 0.27	19.41	19.47	99.38	39.85
Grain length (mm)	5.95–9.30	7.28 ± 0.05	16.55	16.56	99.84	34.06
Grain breadth (mm)	2.09–2.87	2.41 ± 0.02	9.68	9.73	99.00	19.84
Grain length/breadth ratio	2.08–4.30	3.04 ± 0.04	18.86	18.90	99.58	38.77
Decorticated grain length (mm)	4.13–6.97	5.18 ± 0.04	16.99	17.01	99.83	34.98
Decorticated grain breadth (mm)	1.89–2.57	2.20 ± 0.02	8.54	8.57	99.16	17.51
Decorticated grain length/breadth ratio	1.79–2.32	2.38 ± 0.03	19.61	19.63	99.70	40.33
Biological yield (g Plant^−1^)	28.98–44.97	33.56 ± 3.30	8.23	12.83	41.19	10.88
Grain yield (g Plant^−1^)	8.64–17.20	12.94 ± 1.65	9.83	16.09	37.36	12.38
Harvest index (%)	22.99–36.40	31.65 ± 3.16	9.08	13.49	45.26	12.58
Grain yield (kg ha^−1^)	1000.00–3011.67	2012.08 ± 68.16	24.62	24.85	98.14	50.24

### Genetic divergence among the twenty *Joha* rice cultivars

The *V* statistics and the analysis of dispersion (Supplementary Table S6) showed that the mean differences for the pooled effect of the twenty-two characters between the cultivars were highly significant. Mahalanobis distances (*D*^*2*^) distinguished the twenty *Joha* rice cultivars into seven clusters, showing eight, five and three cultivars in I, IV and II, respectively and the rest four with single genotypes (Table 5).The average intra- and inter-cluster distances (Table 6) showed cluster I to have the maximum intra-cluster space (331.08), followed by IV (307.84) and II (223.91). The inter-cluster *D*^*2*^ values varied from 353.94 units between clusters V and VI to 7303.31 between IV and VI. The most distantly related cluster pair IV-VI was followed by IV-V (5187.67), II-IV (4709.44), VI-VII (4073.37), V-VII (2737.52), III-IV (2323.71) and I-IV (2281.86).

**Table 5:** Composition of the Tocher’s clusters basedon Mahalanobis *D*^2^ analysis.

Cluster identity	No. of cultivars	Name of the cultivars
I	8	*Kon Joha (Moran), Jeera Joha, Manimuni Joha, Kon Joha 1, Kon Joha (Bongaigaon), Kon Joha 4, Kalijeera, Kon Joha 2*
II	3	*Keteki Joha, Local Joha, Kon Joha 3*
III	1	*Joha (Bihpuria)*
IV	5	*Ronga Joha, Kola Joha, Joha (Golaghat), Kon Joha (Teok), Harinarayan*
V	1	*Kunkuni Joha*
VI	1	*Kon Joha 5*
VII	1	*Soru Joha (Tinsukia)*

**Table 6:** Intra-(bold) and inter-cluster distances.

Cluster	I	II	III	IV	V	VI	VII
I	**331.08**	1293.16	490.87	2281.86	854.97	1815.30	1167.98
II	1293.16	**223.91**	564.34	4709.44	892.56	1187.69	1560.50
III	490.87	564.34	**0.00**	2323.71	1046.91	1930.59	434.10
IV	2281.86	4709.44	2323.71	**307.84**	5187.67	7303.31	1256.85
V	854.97	892.56	1046.91	5187.67	**0.00**	353.94	2733.52
VI	1815.30	1187.69	1930.59	7303.31	353.94	**0.00**	4073.37
VII	1167.98	1560.50	434.10	1256.85	2733.52	4073.37	**0.00**

The cluster mean performances for the various traits showed variations among the groups (Table 7**)**. Cluster III registered the earliest days to first and 50 per cent flowering (108.33 & 114.17) and maturity (146.57) and the highest mean performance for stem thickness (4.59 mm), flag leaf breadth (0.84 cm), grain yield plant^−1^ (14.83 g) and harvest index (36.40%). The highest values of flag leaf length (54.21 cm), flag leaf area (32.42 cm^2^), panicle length (27.26 cm) and biological yield plant^−1^ (36.73 g) were evident in cluster IV. Mean filled grain panicle^−1^ was the highest (252.07) in the solitary cluster VI, along with grain and decorticated grain breadth (2.87 & 2.33 mm), whereas cluster II registered the shortest height (110.57 cm), the highest number of productive tillers (12.60) and the widest grain length/breadth ratio (3.89). Again, the single cultivar *Soru Joha (Tinsukia)* in cluster VII had the highest means for flag leaf breadth (0.84 cm), spikelet fertility (90.92%), 1000-grain weights (22.50 g), grain/decorticated grain length (9.04/6.97 mm), decorticated grain length-breadth ratio (3.32) and grain yield ha^−1^ (3011.67 kg). Only eight of the twenty-three traits contributed to the genetic divergence. The contribution towards the total variation was the maximum for flag leaf length (72.11%), followed by decorticated grain length (13.68%), grain length (6.84%), decorticated grain breadth (3.16%), grain yield ha^−1^ (1.58%) and grain breadth (1.05%).

**Table 7:** Cluster mean for the traits and their contribution to the total variation.

Character	I	II	III	IV	V	VI	VII	% Contribution to total variation
Stem thickness (mm)	4.15	3.71	4.59	3.98	4.00	4.62	4.28	0.00
Days to 1st flowering	114.98	119.78	108.33	111.53	113.67	118.00	113.33	0.00
Flag leaf length (cm)	47.17	43.63	46.68	54.21	42.80	40.84	49.62	72.11 (1)
Flag leaf breath (cm)	0.77	0.79	0.84	0.80	0.76	0.79	0.84	0.00
Flag leaf area (cm^2^)	27.16	25.76	29.20	32.42	24.53	24.21	31.13	0.00
Days to 50% flowering	119.79	125.67	114.17	116.67	119.33	126.33	117.50	0.00
Days to maturity	151.69	158.69	146.57	149.04	151.73	160.60	149.50	0.00
Plant height (cm)	131.44	110.57	135.60	131.21	134.43	122.77	124.03	0.00
Productive tillers Plant^−1^	9.56	12.60	9.73	11.20	9.37	8.77	10.23	0.00
Panicle length (cm)	25.76	25.80	26.64	27.26	24.34	25.19	26.79	0.00
Filled grains Panicle^−1^	207.15	139.80	161.83	145.06	198.90	252.07	129.50	0.00
Spikelet fertility (%)	87.86	83.36	89.90	88.03	87.03	79.89	90.92	0.00
1000-grain weights (g)	15.65	19.19	18.22	19.84	13.02	16.31	22.50	1.05 (6)
Grain length (mm)	6.41	8.93	8.12	7.67	6.08	5.95	9.04	6.84 (3)
Grain breadth (mm)	2.41	2.32	2.29	2.45	2.19	2.87	2.42	1.05 (6)
Grain length/breadth ratio	2.67	3.89	3.54	3.13	2.78	2.08	3.74	0.00
Decorticated grain length (mm)	4.60	6.17	6.15	5.35	4.29	4.17	6.97	13.68 (2)
Decorticated grain breadth (mm)	2.20	2.07	2.16	2.29	2.09	2.33	2.10	3.16 (4)
Decorticated grain length/breadth ratio	2.11	3.00	2.85	2.33	2.05	1.79	3.32	0.53 (7)
Biological yield (g Plant^−1^)	31.71	33.30	34.31	36.73	32.71	34.96	31.97	0.00
Grain yield (g Plant^−1^)	12.12	14.12	14.83	13.72	11.74	11.65	12.57	0.00
Harvest index (%)	31.57	32.96	36.40	30.81	28.51	28.70	33.89	0.00
Grain yield (kg ha^−1^)	2029.38	1715.56	2621.67	1799.00	2378.33	1853.33	3011.67	1.58 (5)
No. of traits with the highest mean	0	3	7	4	0	3	6	

**Figure 3:**
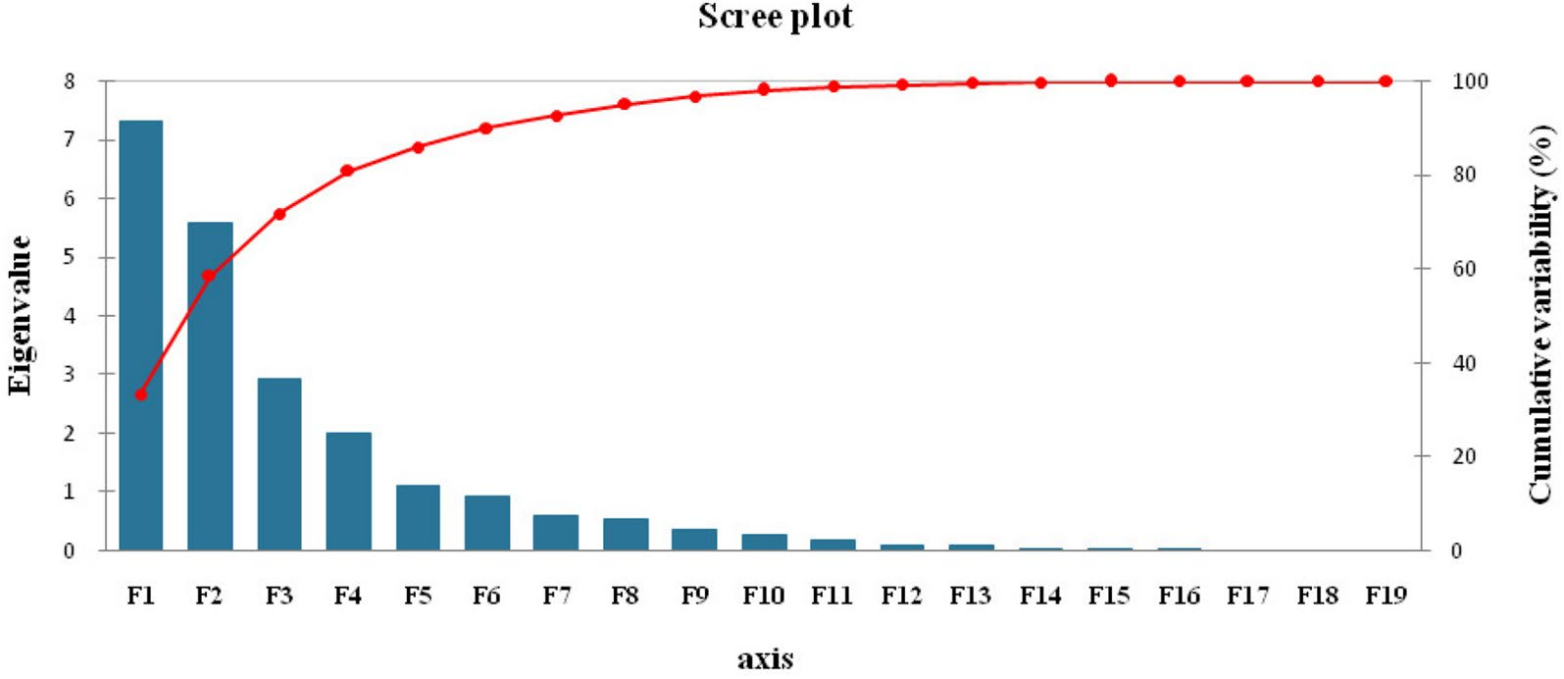
Scree plot showing Eigen values and percentage of cumulative variability.

**Figure 4:**
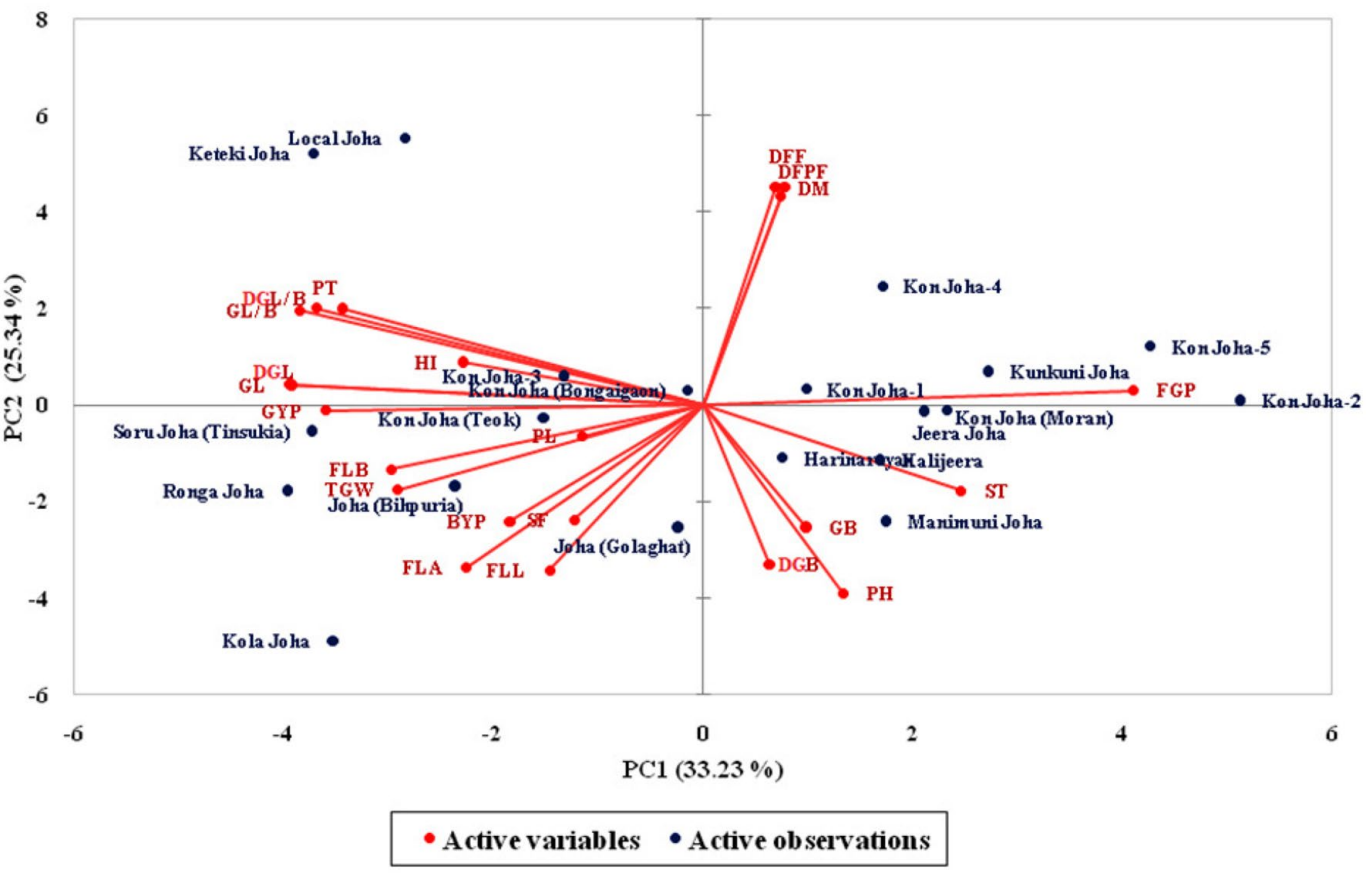
Distribution of 20 indigenous *Joha* rice cultivars and 22 traits across first two components based on PCA.

Principal component analysis (PCA) was performed for twenty-two traits of the 20 indigenous *Joha* rice cultivars (Supplementary Table S7). Principal components (PCs) assume importance when the eigenvalue is greater than one and the PC explains at least 5% of the variation in the data^32^. Out of twenty-one, only five principal components (PCs) exhibited eigenvalue more than one and explained 85.87% cumulative variability among the traits studied; thus, these five PCs were significant for further explanation. The first five PCs explained 33.23, 25.34, 13.23, 9.11 and 4.95% of the variability among the cultivars for the traits under study. The Scree plot (Fig. 3) showed slight variance after the fifth PC. The traits filled grains panicle^−1^ (0.91) and stem thickness (0.55) positively contributed to the first PC. In contrast, decorticated grain length (-0.88), grain length (-0.87), grain length/breadth ratio (-0.85), decorticated grain length/breadth ratio (-0.820) and grain yield plant^−1^ (-0.80) contributed negatively to PC 1. PC 2 accounted for 25.34% of the total variability. The positively related traits were days to 50% flowering (0.88), days to maturity (0.88) and days to first flowering (0.84), while plant height (-0.76), flag leaf length (-0.67) and flag leaf area (-0.65) were negatively related to PC 2. PC 3 contributed 13.23% to the total variability, with grain breadth (0.76), 1000-grain weights (0.60), decorticated grain breadth (0.59), and biological yield plant^−1^ (0.52) having a positive contribution, while the harvest index (-0.56) and spikelet fertility (-0.55) contributed negatively to PC 3. PC 4 and PC 5 contributed 9.11 and 4.95% of the total variability, respectively. The vector length depends on the character's contribution to the principal component (Fig. 4). Moreover, the vectors' angle reflects the variables' correlation. If the angle between two trait vectors is < 90° (an acute angle), it indicates a positive correlation. The vectors in the first quadrant, viz., days to first/ 50% flowering and maturity, strongly correlated among themselves and loaded on the PC2. At the same time, filled grains panicle^−1^ loaded on the PC1 had a weak correlation with the above traits. The vectors in the second quadrant, productive tillers, decorticated grain length-breadth ratio, grain length-breadth ratio, decorticated grain length and grain length, were highly correlated variables loaded on PC1. Similarly, the vectors in the third quadrant, grain yield plant^−1^, panicle length, flag leaf breadth, 1000-grain weights and flag leaf area, were highly correlated variables and loaded on PC1. In the fourth quadrant, stem thickness loaded on PC1 and grain/decorticated grain breadth and plant height correlated to PC2; the latter three vectors were also highly interrelated. If the angle between two traits is > 90° (an obtuse angle), it indicates a negative correlation, while if the grade is equivalent to 90°, it suggests no correlation between the traits. The traits stem thickness, filled grains panicle^−1^, days to first flowering, grain breadth, plant height and days to 50% flowering were negatively correlated with grain yield plant^−1^. The cultivar *Soru Joha* (*Tinsukia*) projected on the vectors of productive tillers plant^−1^, decorticated grain length/breadth ratio, grain length/breadth ratio, decorticated grain length, grain length and grain yield plant^−1^ were close to them, indicating a positive interaction (Fig. 4). Comparing the twenty cultivars, the cultivar *Joha (Bihpuria)* was superior for flag leaf breadth, 1000-grain weights, biological yield plant^−1^, flag leaf area, flag leaf length, spikelet fertility, panicle length and grain yield plant^−1^. Moreover, the cultivars *Kon Joha (Teok)*, *Soru Joha (Tinsukia)*, *Ronga Joha*, *Joha (Golaghat)* and *Kola Joha* also had a positive interaction with those characters. The cultivars with a high positive principal component score for PC 1 (Supplementary Table S8) were *Keteki Joha* (1.912), *Local Joha* (1.636), *Soru Joha* (*Tinsukia*) (1.273), *Ronga Joha* (1.219), *Kola Joha* (0.717) and *Joha Bihpuria* (0.660).

### Biochemical characterization of the *Joha* rice cultivars

Table 8 shows the biochemical characterization of the twenty *Joha* rice cultivars based on fatty acid profile, Fe and Zn content, crude protein, amylose, gel consistency, and aroma. A cultivar's mean value was considered desirable for all other biochemical traits except for polyunsaturated fatty acids when it exceeded the cultivars' mean plus the standard deviation. A low mean less than the cultivars' mean minus the standard deviation was desirable for polyunsaturated fatty acids.

**Table 8:** Biochemical characteristics of the twenty *Joha* rice cultivars.

Cultivars	Palmitic acid (16:0)	Stearic acid (18:0)	Oleic acid (18:1)	Linoleic acid (18:2)	Linolenic acid (18:3)	Arachidic acid (20:0)	Fe (mg kg^−1^)	Zn (mg kg^−1^)	Protein content (%)	Amylose content (%)	Gel consistency (mm)	Aroma (KOH test score)
Joha (Bihpuria)	19.45	1.29	37.93	37.91	1.09	0.38	23.41	26.73	8.94	20.10	140.50	2
Kalijeera	18.50	1.86	40.70	36.77	1.23	0.61	31.19	23.49	9.69	17.40	84.50	3
Ronga Joha	18.22	1.25	38.55	39.62	1.33	0.54	47.00	47.39	7.51	19.10	113.00	1
Joha (Golaghat)	19.16	1.50	41.32	36.02	1.11	0.51	28.58	32.60	7.93	19.42	129.50	2
Manimuni Joha	18.97	1.59	40.56	36.51	1.36	0.57	36.23	26.71	9.37	23.20	121.50	3
Kon Joha (Moran)	19.19	1.48	36.90	39.83	1.66	0.54	25.61	28.56	9.87	17.30	98.00	2
Keteki Joha	18.08	1.20	37.37	40.90	1.77	0.37	21.33	12.26	9.07	22.60	82.00	2
Kon Joha 1	18.19	1.70	38.66	38.92	1.50	0.61	61.00	17.50	10.32	16.20	105.50	2
Soru Joha (Tinsukia)	18.78	1.59	40.23	37.32	1.09	0.59	61.09	22.98	8.20	19.50	94.50	2
Kon Joha-2	19.21	1.56	38.07	38.23	2.10	0.51	62.50	23.00	9.89	22.60	86.00	3
Jeera Joha	18.86	1.19	35.71	41.82	1.63	0.43	43.07	37.95	8.59	15.20	118.50	2
Kon Joha-3	19.18	1.40	40.19	36.30	1.86	0.61	24.05	45.32	8.65	21.40	79.50	2
Kon Joha-4	20.11	1.50	37.91	38.26	1.24	0.55	82.88	38.25	9.23	21.40	71.00	2
Kunkuni Joha	19.78	1.04	33.53	43.13	1.78	0.40	34.61	28.76	9.17	15.70	123.00	3
Kon Joha-5	18.94	1.72	39.84	36.72	1.93	0.50	34.91	24.45	8.41	18.92	61.50	3
Local Joha	17.98	1.08	33.66	44.61	2.14	0.28	51.91	20.13	9.38	23.84	85.50	2
Harinarayan	19.94	0.90	35.56	41.14	1.81	0.29	53.83	31.30	9.18	24.40	91.00	2
Kon Joha (Teok)	20.57	1.10	34.92	41.41	1.34	0.34	63.66	31.80	8.66	18.00	113.50	1
Kola Joha	18.76	1.63	40.26	37.36	1.06	0.53	37.31	25.86	10.20	17.20	95.50	2
Kon Joha (Bongaigaon)	18.38	1.48	36.10	41.58	1.63	0.49	47.21	23.62	9.65	23.80	111.00	2
Lowest mean	17.98	0.90	33.53	36.02	1.06	0.28	21.33	12.26	7.51	15.20	61.50	1
Highest mean	20.57	1.86	41.32	44.61	2.14	0.61	82.88	47.31	10.32	24.40	140.50	3
Mean	19.01	1.40	37.90	39.22	1.53	0.48	43.57	28.43	9.09	19.86	100.25	2.15
SE	0.16	0.06	0.53	0.56	0.08	0.02	3.75	1.96	0.35	0.56	3.23	0.13
CV (%)	3.71	18.49	6.30	6.35	22.53	22.01	38.51	30.76	8.22	14.62	20.72	27.3

### Fatty acid profile

Palmitic acid content ranged from 17.98 (*Local Joha*) to 20.57 per cent (*Kon Joha-Teok*), averaging 19.01 (Table 8). The stearic acid content was the lowest (0.90%) in *Harinarayan* and the highest (1.86%) in *Kalijeera*; the standard was 1.40. The range of oleic acid was from 33.53 per cent in *Kunkuni Joha* to 41.32 per cent in *Joha (Golaghat)*, with an average of 37.90. The linoleic acid content varied from 36.02 in *Joha (Golaghat) *to 44.61% in *Local Joha,* with an average of 39.22. *Local Joha* recorded the highest linolenic acid content (2.14%), whereas *Kola Joha* had the lowest estimate (1.06%). The arachidic acid content ranged from 0.28 (*Local Joha*) to 0.61 *per cent (Kon Joha-3*), with an average of 0.48.

### Iron and Zn content of the cultivars

The iron content in the cultivars varied from 21.33 (*Keteki Joha*) to 82.88 mg kg^−1^ in *Kon Joha 4*, with an average of 43.57 mg kg^−1^ (Table 8). *Kon Joha 4* was followed by *Kon Joha-Teok* (63.66 mg kg^−1^), *Kon Joha 2* (62.50 mg kg^−1^), *Soru Joha-Tinsukia* (61.09 mg kg^−1^) and *Kon Joha 1* (61.00 mg kg^−1^).

The zinc content ranged from 12.26 (*Keteki Joha*) to 47.39 mg kg^−1^ in *Ronga Joha*, showing an average of 28.43 mg kg^−1^. The other cultivars having high grain zinc content were* Kon Joha-3* (45.32 mg kg^−1^), *Kon Joha-4* (38.25 mg kg^−1^) and *Jeera Joha* (37.95 mg kg^−1^).

### Protein content, amylose content, gel consistency, and aroma score of the cultivars

The cultivars' protein content ranged from 7.51 per cent in *Ronga Joha* to 10.32 per cent in *Kon Joha-1,* with an average of 9.09 per cent (Table 8). The amylose content varied from 15.20 in *Jeera Joha* to 24.40% in *Harinarayan*, with an average of 19.86. The cultivars exhibited two classes of amylose content*—*medium (20–25%) and low (10–20%). The lowest and the highest gel consistency in the cultivars were 61.50 in *Kon Joha-5* and 140.50 mm in *Joha (Bihpuria)*, respectively*, *with an average of 100.25. *Joha-Golaghat* (129.50 mm), *Kunkuni Joha* (123.00 mm), and *Manimuni Joha* (121.50 mm) followed *Joha (Bihpuria)*. The cultivars viz., *Kalijeera, Kunkuni Joha, Kon Joha-5, Manimuni Joha, *and *Kon Joha-2* showed a strong aroma, and *Joha (Bihpuria), Keteki Joha, Kon Joha-3, Kon Joha (Moran), Joha (Golaghat), Kola Joha, Jeera Joha, Kon Joha (Bongaigaon), Local Joha, Harinarayan, Kon Joha-1, Soru Joha (Tinsukia) and Kon Joha-4* registered a mild aroma. Ronga *Joha* and *Kon Joha (Teok)* possess a light scent.

### Molecular characterization

**Figure 5:**
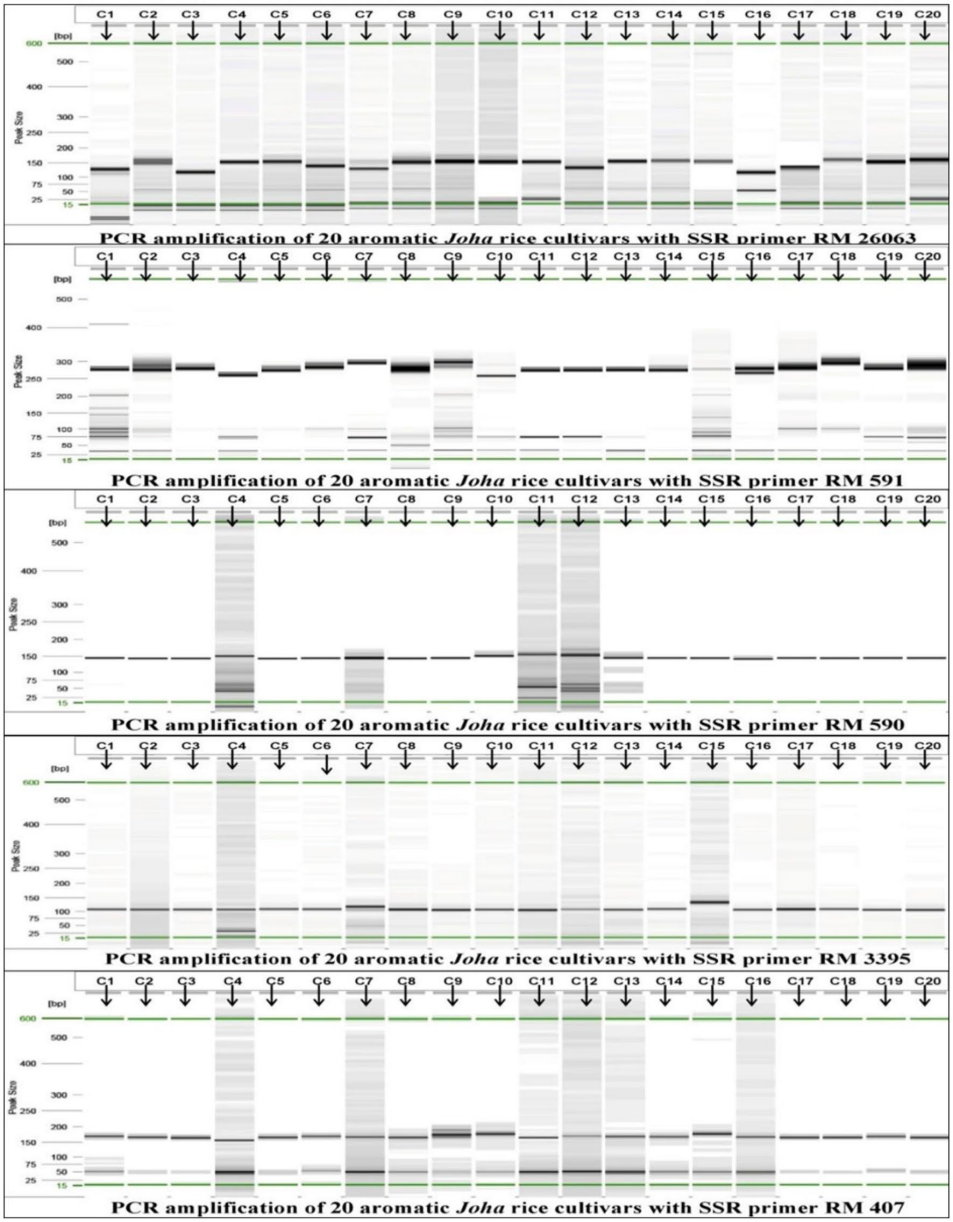
Representative gel pictures showing the PCR products.

Among the seventy-one SSR markers, sixty-six showed polymorphisms. The analysis excluded markers with monomorphic banding patterns. Table 9 summarizes the results on twenty aromatic rice cultivars using the polymorphic SSR loci. Figure 5 depict representative gel pictures of the PCR products.

**Table 9:** SSR markers profile of the twenty indigenous *Joha* rice cultivars.

Locus	*Na*	*MAF*	*Ne*	*I*	*Ho*	*He*	*PIC*
RM495	2	0.90	1.220	0.325	0.00	0.180	0.164
RM 283	4	0.40	3.509	1.320	0.00	0.715	0.665
RM 237	2	0.95	1.105	0.199	0.00	0.095	0.091
RM431	3	0.70	1.802	0.746	0.00	0.445	0.381
RM154	3	0.70	1.802	0.746	0.00	0.445	0.381
OSR 13	3	0.80	1.504	0.613	0.00	0.335	0.303
RM338	2	0.65	1.835	0.647	0.00	0.455	0.352
RM514	3	0.60	2.062	0.824	0.00	0.515	0.352
RM124	2	0.90	1.220	0.325	0.00	0.180	0.164
RM 161	2	0.55	1.980	0.688	0.00	0.495	0.373
RM133	3	0.75	1.681	0.731	0.00	0.405	0.368
RM 125	3	0.85	1.361	0.518	0.00	0.265	0.247
RM 118	4	0.55	2.469	1.070	0.00	0.595	0.531
RM 152	2	0.85	1.342	0.423	0.00	0.255	0.352
RM284	3	0.85	1.361	0.518	0.00	0.265	0.247
RM316	4	0.30	3.922	1.376	0.00	0.745	0.698
RM 215	2	0.95	1.105	0.199	0.00	0.095	0.091
RM 271	2	0.88	1.280	0.377	0.05	0.219	0.164
RM484	2	0.85	1.342	0.423	0.00	0.255	0.352
RM 536	2	0.50	2.000	0.693	0.00	0.500	0.375
RM 227	2	0.60	1.923	0.673	0.00	0.480	0.365
RM 29	3	0.45	2.410	0.949	0.00	0.585	0.495
RM489	3	0.70	1.802	0.746	0.00	0.456	0.381
RM55	3	0.85	1.361	0.518	0.00	0.272	0.247
RM 510	2	0.55	1.980	0.688	0.00	0.508	0.373
RM474	3	0.60	2.247	0.938	0.00	0.569	0.491
RM 171	3	0.45	2.740	1.049	0.00	0.651	0.559
RM212	2	0.90	1.220	0.325	0.00	0.185	0.164
RM23	3	0.60	2.062	0.824	0.00	0.528	0.424
RM 229	2	0.70	1.724	0.611	0.00	0.431	0.332
RM1003	2	0.70	1.724	0.611	0.00	0.431	0.332
RM29	4	0.60	2.299	1.033	0.00	0.579	0.509
RM 221	2	0.80	1.471	0.500	0.00	0.328	0.269
Mean	2.636	0.727	1.759	0.640	0.003	0.387	0.326
SE	0.079	0.020	0.072	0.035	0.001	0.022	0.018
RM6641	2	0.95	1.105	0.199	0.00	0.097	0.091
RM 279	2	0.88	1.280	0.377	0.05	0.224	0.091
RM3562	3	0.45	2.740	1.049	0.00	0.651	0.559
RM60	2	0.65	1.835	0.647	0.00	0.467	0.352
RM1388	3	0.45	2.817	1.067	0.00	0.662	0.572
RM471	2	0.85	1.342	0.423	0.00	0.262	0.223
RM17467	3	0.70	1.869	0.819	0.00	0.477	0.420
RM 317	2	0.85	1.342	0.423	0.00	0.262	0.223
RM 187	2	0.85	1.342	0.423	0.00	0.262	0.223
RM 3322	2	0.90	1.220	0.325	0.00	0.185	0.164
RM 585	4	0.50	2.740	1.142	0.00	0.651	0.573
RM 20236	2	0.90	1.220	0.325	0.00	0.185	0.164
RM 7434	3	0.78	1.578	0.652	0.05	0.376	0.303
RM2126	2	0.90	1.220	0.325	0.00	0.185	0.164
RM 253	3	0.55	2.151	0.845	0.00	0.549	0.436
RM217	2	0.90	1.220	0.325	0.00	0.185	0.164
RM434	3	0.85	1.361	0.518	0.00	0.272	0.247
RM 481	3	0.70	1.852	0.802	0.00	0.472	0.410
RM11	3	0.55	2.410	0.975	0.00	0.600	0.513
RM 505	3	0.65	1.942	0.791	0.00	0.497	0.406
RM501	3	0.73	1.751	0.749	0.05	0.440	0.347
RM25	3	0.85	1.361	0.518	0.00	0.272	0.247
RM407	3	0.70	1.852	0.802	0.00	0.472	0.410
RM3481	2	0.95	1.105	0.199	0.00	0.097	0.091
RM3395	3	0.90	1.227	0.394	0.00	0.190	0.177
RM228	3	0.85	1.361	0.518	0.00	0.272	0.247
RM590	2	0.80	1.471	0.500	0.00	0.328	0.269
RM591	3	0.70	1.852	0.802	0.00	0.472	0.410
RM 26063	4	0.60	2.381	1.089	0.00	0.595	0.438
RM21	2	0.95	1.105	0.199	0.00	0.097	0.091
RM27601	3	0.90	1.227	0.394	0.00	0.190	0.177
R1M7	2	0.85	1.342	0.423	0.00	0.262	0.223
R1M30	3	0.45	2.597	1.010	0.00	0.631	0.534
Mean	2.636	0.727	1.759	0.640	0.003	0.387	0.326
SE	0.079	0.020	0.072	0.035	0.001	0.022	0.018

*Na*: Number of different alleles amplified; *MAF*: Major allele frequency; *Ne*: Number of effective alleles; *I*: Shannon’s informative index; *Ho*: Observed heterozygosity; *He*: Expected heterozygosity; *PIC*: Polymorphism information content.

The 66 polymorphic SSR loci amplified a total of 174 alleles (Supplementary Table S9). The allelic richness per locus was 2 to 4, with an average of 2.64 alleles. Among the polymorphic markers, 30 produced two alleles each, 30 produced three alleles each, and 6 generated four alleles. The markers RM283, RM118, RM316, RM29, RM585, and RM26063 amplified the maximum number of alleles. The results revealed that all the markers showed distinct polymorphisms among the cultivars studied, indicating the robust nature of microsatellites revealing polymorphisms.

Among the sixty-six markers, the highest major allele frequency was 0.950 in five (RM237, RM215, RM6641, RM3481 & RM21), followed by 0.900 in nine and 0.875 in two markers (Table 9).The lowest major allele frequency was 0.300 for RM316. The mean value of major allele frequency was 0.727, ranging from 0.300 to 0.950 among the sixty-six markers scored against the twenty *Joha* rice cultivars. The number of effective alleles ranged from 1.105 (RM237) to 3.922 (RM316), with a mean value of 1.759. Shannon's information index ranged from 0.199 (RM237) to 1.376 (RM316), with a mean value of 0.640. The gene diversity varied from 0.095 to 0.745, with a mean value of 0.377. RM316 showed the highest genetic diversity (0.745), followed by RM283 (0.715) and RM1388 (0.645). A set of four markers, viz., RM271, RM279, RM7434, and RM501, represented heterozygosity among the sixty-six SSRs. All four SSR markers showed the same value, 0.050, with a mean value of 0.003. The rest markers showed no heterozygosity. The polymorphism information content (PIC) values, a reflection of the allelic diversity and frequency among the genotypes, also varied from one locus to another (Table 9). The PIC values ranged from 0.091 to 0.698, with an average of 0.326, indicating that only some SSR markers were highly informative. The highest PIC value of 0.698 was obtained for RM316, followed by RM283 (0.665), RM585 (0.573), RM1388 (0.572), RM3562 (0.559), RM171 (0.559), R1M30 (0.534), RM118 (0.531), RM11 (0.513) and RM29 (0.509), suggesting that these ten markers were highly informative (PIC > 0.50). Among the remaining polymorphic SSR markers, thirty were informative (0.50 < PIC > 0.25), and twenty-six were slightly informative (PIC < 0.25).

An allele observed in only one of the 20 *Joha* rice cultivars was considered unique. Twenty-seven of the 66 polymorphic SSR loci detected 28 private alleles (97–362 bp) specific to 13 cultivars, with an average of 1.04 per locus. RM3395 amplified a maximum of two unique alleles, followed by the others with single alleles (Table 10). The cultivar *Kon Joha-5 *had nine unique alleles, followed by *Kon Joha-4* (4), *Kon Joha-2 *(3), *Ronga Joha *and *Joha (Bihpuria),* each with two alleles. *Jeera Joha*, *Kon Joha (Moran)*, *Kola Joha*, *Harinarayan*, *Kalijeera*, *Keteki Joha*, *Kon Joha-3* and *Kon Joha (Bongaigaon)* showed single private alleles.

**Table 10:** Unique SSR alleles specific to the aromatic *Joha* rice cultivars.

SSR marker	Chr. No	Allele size (bp)	Cultivars with unique allele
RM431	1	260	*Kon Joha-5*
R1M30	1	197	*Ronga Joha*
RM6641	2	183	*Jeera Joha*
RM154	2	200	*Kola Joha*
RM514	3	179	*Joha (Bihpuria)*
RM489	3	160	*Kon Joha-4*
OSR13	3	117	*Kon Joha-5*
RM55	3	245	*Kalijeera*
RM253	6	131	*Kon Joha-5*
RM585	6	184	*Harinarayan*
RM7434	6	139	*Kon Joha-5*
RM125	7	175	*Kon Joha-4*
RM118	7	362	*Kon Joha-4*
RM481	7	145	*Kon Joha-4*
RM505	7	198	*Joha (Bihpuria)*
RM25	8	150	*Kon Joha-5*
RM3395	8	130	*Kon Joha-5*
RM3395	8	122	*Keteki Joha*
RM3481	8	200	*Kon Joha-5*
RM215	9	97	*Kon Joha (Moran)*
RM434	9	138	*Kon Joha-5*
RM228	10	158	*Kon Joha-2*
RM21	11	174	*Kon Joha-2*
RM27601	12	150	*Kon Joha-5*

### Molecular diversity among the aromatic rice cultivars

**Figure 6:**
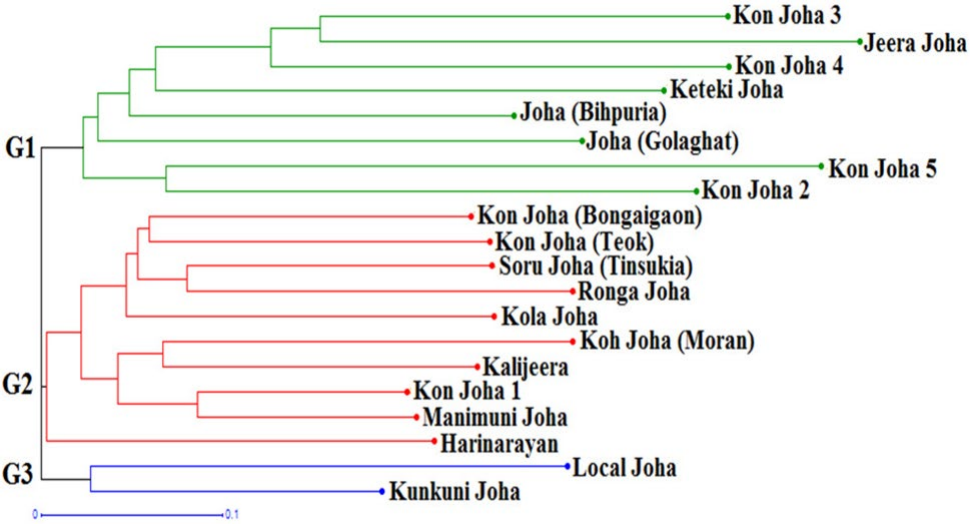
Hierarchical horizontal clustering of the 20 *Joha* rice cultivars using Unweighted Neighbour-Joining (UNJ) method based on Jaccard’s coefficients of similarity estimated from 66 polymorphic SSR markers.

An unweighted neighbour-joining (UNJ) cluster analysis based on Jaccard's dissimilarity coefficients (Supplementary Table S10) resolved the phylogenetic relationships among the *Joha* rice cultivars collected from different parts of Assam. The UNJ cluster diagram showed three major clusters (G1, G2 & G3) with additional sub-clusters (Fig. 6); the dendrogram revealed that the cultivars derived from a genetically similar type clustered together. Cluster I comprised eight cultivars, whereas cluster II had ten cultivars, forming the most significant group. Cluster III had only two cultivars (*Local Joha* & *Kunkuni Joha*). Cluster I included *Kon Joha-3*, *Jeera Joha*, *Kon Joha-4*, *Keteki Joha*, *Joha (Bihpuria)*, *Joha (Golaghat)*, *Kon Joha-5*, and *Kon Joha-2*. *Kon Joha (Bongaigaon)*, *Kon Joha (Teok)*, *Soru Joha (Tinsukia)*, *Ronga Joha*, *Kola Joha*, *Kon Joha (Moran)*, *Kalijeera*, *Kon Joha-1*, *Manimuni Joha* and *Harinarayan* belonged to cluster II. Cluster I was subdivided into two groups, IA and IB, consisting of six and two cultivars, respectively. Cluster II had two sub-clusters, IIA and IIB, composed of nine and one cultivars, respectively.

## Discussion

Qualitative characteristics are the morphological markers for identifying rice landraces because environmental changes least influence these traits^33^. The thirty-seven stable morphological characteristics would serve as reliable morphological markers for identifying the twenty *Joha* rice cultivars, corroborating earlier studies^34^. A substantial amount of variability within this specialty class of rice was evident from the observed clustering pattern based on the morphological markers, which agrees with Mondal et al.^35^.

Significant cultivar differences in aromatic rice were also reported in earlier studies [36 &37]. The phenotypic expression of most yield and contributing traits differed significantly in the 2018 and 2019 crops; the 17 June planted crop in 2019 exhibited higher mean performances than that of the 11 July planted crop in 2018. Delaying the sowing time decreased the days to flowering and maturity for most cultivars. A similar observation was reported by Song et al.^38^ for days to heading reduced in different rice cultivars due to delayed sowing. Nahar et al.^39^ observed a significant decrease in filled grain production consequent to delayed transplanting, attributed to low temperature at anthesis and primordial spikelet formation. Khalifa^40^ noted the early date of sowing as the best time for maximizing morpho-physiological traits such as tillering, panicle initiation, chlorophyll content, leaf area index, sink capacity, panicle length, panicle number, and grain yield. Delayed sowing significantly reduces the number of filled grains, panicles, and test weight, finally lowering rice cultivars' grain yield^41^. Nevertheless, a significant year x cultivar interaction component for days to flowering and maturity, filled grain panicle^−1^, and spikelet fertility suggested variation in adaptive traits among the *Joha* rice cultivars. Panwar et al.^42^ also reported substantial year x cultivar interactions for days to 50% flowering and days to maturity.

The low tillering habit of the aromatic rice cultivars was also supported by Singh et al.^43^. The considerable genetic variability for yield components observed in the present study was also similar to the findings of Singh et al.^43^. The variations in the grain characteristics of the *Joha* rice cultivars were consistent with the findings of Singh et al.^43^ and Semwal et al.^44^. The findings of Bajpai and Singh^45^ further corroborated the present results on grain physical quality characteristics. The phenotypic variations for all the traits except productive tillers plant^−1^ were mainly determined by the genotypes, indicating the effectiveness of phenotype-based selection. These findings were in line with Karim et al.^46^. Earlier reports also support the present results on broad sense heritability for flowering/maturity duration, flag leaf and grain characteristics. Chavan et al.^47^ obtained high heritability for days to 50% flowering (97.5%), plant height (99.5%), filled grains panicle^−1^ (99.7%), test weight (96.8%) and kernel length (98.7%) in aromatic rice. Debsharma et al.^48^ also recorded high heritability for days to 50% flowering, ranging from 97.9 to 99.4% in rice genotypes tested over three locations. Similarly, Akshay et al.^49^ reported high heritability in rice for days to 50% flowering (96.1%), plant height (98.6%), grains panicle^−1^ (97.8%), 1000-grain weights (99.35), length (98.1%), width (99.4%) and length/breadth ratio (98.7%) of grains. In transplanted aman rice, Faysal et al.^50^ found high heritability for days to 50% flowering (98.46%), plant height (99.26%), flag leaf length (99.83%), and 1000-grain weights (99.49%).

The traits excluding days to first/50 per cent flowering and maturity, flag leaf breadth, and spikelet fertility exhibited high heritability in conjunction with high to moderate genetic advance, indicating the most likely role of additive gene action and effectiveness of simple selection for the traits. High heritability and low genetic advance for days to 50% flowering agreed with Chaurasia et al.^51^. Plant height registered high heritability and moderate genetic advancement in conformity with the findings of Chaurasia et al.^51^. Moderate heritability and high genetic advance for productive tillers plant^−1^ were consistent with Jaiswal et al.^52^. Filled grains panicle^−1^ exhibited a high heritability concomitant with high genetic advance, in agreement with the results of Hasib et al.^53^. High heritability in concurrence with high genetic advance for 1000-grain weights was in accordance with the findings of Nandan et al.^54^. The grain quality traits viz., grain length, breadth and length-breadth ratio, decorticated grain length, breadth, and length-breadth ratio registered high heritability coupled with the high genetic advance in consonance with the findings of Jaiswal et al.^52 ^for grain length, grain breadth and length-breadth ratio. A low heritability coupled with moderate genetic advance for grain yield plant^−1^ was in agreement with Adjah et al.^55^.

Mahalanobis distance-based clustering pattern of the twenty *Joha rice* cultivars into seven groups confirmed the quantum of diversity present in the indigenous aromatic rice of Assam and offered scope for its exploitation through breeding for yield improvement. Previous studies reported different numbers of clusters in fragrant rice, *e.g*., six by Allam et al.^56^ and five groups by Patel et al.^57^. Prasad et al.^58^ obtained 20 Euclidean distance-based clusters in 208 Indian aromatic rice accessions, with 57 genotypes in the largest and single genotypes in six groups. Barhate et al.^59^ used Mahalanobis D^2^ statistics to classify 45 aromatic rice genotypes into 10 clusters; five had single genotypes. Netam et al.^60^ classified 40 scented rice genotypes into five groups, the largest having 29 and two with single genotypes. In the present study, cluster pairs viz., IV-VI, IV-V, II-IV, VI-VII, V-VI, and III-IV as widely divergent, and thus, hybridization between parents from these contrasting clusters would likely produce a broad spectrum of variability and transgressive segregations with high heterotic effects, also suggested by Allam et al.^56^ and Patel et al.^57^. Flag leaf length had the highest contribution to total divergence, suggesting the scope for grain yield enhancement through crossbreeding among the aromatic rice cultivars. Since aromatic rice cultivars have the poor combining ability, crossbreeding with non-aromatic varieties would decrease aroma and quality^61^. The rice flag leaf is the main photosynthetic organ crucial in grain yield^62^. The morphological variation in flag leaves directly affects the population structure, light distribution, and energy utilization and is, therefore, an essential target trait in breeding super high-yield hybrid rice^63^. Rice breeders emphasize flag leaf characteristics in selecting the ideal rice phenotype. Rice flag leaves are significant functional leaves for grain filling, and their photosynthesis contributes more than half of all carbohydrates in rice seeds^64^. With its two main components, the flag leaf area is essential in determining its photosynthesis capacity and is influenced by multiple QTLs and their interactions with the environment^62^. Among the other traits, the aromatic rice cultivars’ decorticated grain and grain length significantly varied. These results agreed with the findings of Allam et al.^56^ for decorticated grain length and Singh et al.^65^ for grain length.

The study of many morphological characters in germplasm is vital for the assessment of the differences among populations as well as for the examination of their breeding potential. Plant breeders often measure many variables, some of which may not be of sufficient discriminatory power for germplasm evaluation, characterization and management. In such cases, principal component analysis (PCA) may reveal the patterns and eliminate redundancy in data sets. The PCA, or canonical root analysis, is a multivariate statistical technique to simplify and analyze the relationships among an extensive collection of variables in terms of a relatively small group of components without losing any essential information of the original data set. PCA is a powerful tool to identify the minimum number of components, explain the maximum variability out of the total variability^66^ and rank genotypes based on PC scores. The cumulative variance of 85.87% by the first five axes with an Eigenvalue > 1.00 indicates that the identified traits significantly influenced the cultivars' phenotype and could effectively be used for selection among them. These results corroborated Lakshmi et al.^67^. Burman et al.^68^ reported that four principal components (PCs) exhibited eigenvalues of more than 1.00 and explained 81.62% variability. Ahmed et al.^69^ showed that the first five components with vector values > 1.00 contributed 82.90% of the total variations in 31 rice germplasm lines. Pachauri et al.^70^ studied one hundred twenty-four rice germplasm accessions based on nineteen morphological and eleven agronomic traits. From their studies, PC1 expressed 37.12% variability, while PC2, PC3 and PC4 recorded 13.56, 11.04 and 10.76% variability, respectively, and traits such as the number of effective tillers, 100-grain weights were the principal discriminatory traits. PCA helps us identify the characteristics that significantly impact the phenotype of different rice landraces, which is very important in the selection procedure of the breeding program. The biplot analysis showed the relationships between the morphological traits among the tested genotypes. Acute angles were apparent between productive tillers, decorticated grain length/breadth ratio, grain length/breadth ratio, grain yield plant^−1^, panicle length, flag leaf breadth, 1000-grain weights, and flag leaf area; the selection of these traits would significantly contribute to *Joha* rice improvement. Increased grain yield is associated with 1000-grain weights^71^ and long panicle lengths with many filled grains^72^. The seed size, such as seed length and breadth, also significantly increases the grain yield plant^−1^^72^. The traits influencing PC 1 were flag leaf breadth, 1000-grain weights, flag leaf area, filled grains panicle^−1^, stem thickness, decorticated grain length/breadth ratio, grain length/breadth ratio and grain yield plant^−1^. These results also support the GCV estimates for flag leaf length, filled grains panicle^−1^, 1000-grain weights, decorticated grain length, stem thickness and grain length/breadth ratio; the first three traits, along with stem thickness, also corroborated Mahalanobis distance-based divergence. The cultivars *Soru Joha (Tinsukia)*, *Ronga Joha*, *Joha (Bihpuria)* and *Kola Joha* had a high principal component score for PC 1. Based on the relationship of traits and cultivars to PC 1, the cultivars *Joha (Bihpuria) *and *Soru Joha (Tinsukia)* would serve as parents for the above characteristics for breeding improved *Joha* rice.

Fatty acids are vital components of food and human health. Fatty acids are the major constituents of the cell membrane structure and play important biological, structural, and functional roles in the human body^73^. They act as modulators of gene transcription, cytokine precursors, and energy sources in complex interconnected systems^74^ by producing a vast ATP quantity during their metabolism^73^. The role of dietary fatty acids in human health is strongly evident in their influence on cardiovascular disease and mental health^74^. In addition, rice is a dietary consumption; rice fats have unique health benefits^75^. In the present investigation, oleic, linoleic, and palmitic acids were the primary fatty acids, and stearic, linolenic, and arachidic acids were minor in the aromatic rice cultivars. Palmitic, stearic, and arachidic acids are saturated fatty acids in rice bran, increasing health risks such as atherosclerosis, and associated with a heart attack^76^. Linoleic acid is absorbed as a predominant unsaturated fatty acid, followed by oleic and linolenic acid. High contents of polyunsaturated fatty acids are desirable for human health, as their consumption minimizes the risk of heart-related diseases^77^. The mean polyunsaturated fatty acid (PUFA) contents of the aromatic cultivars were 37.9% for oleic acid, 39.22% for linoleic acid, and 0.5% for linolenic acid, whereas the contents of saturated fatty acids (SFAs) were 1.40% for stearic acid and 19.01% for palmitic acid. These estimates were comparable to or even better than the values of 38.4% oleic acid, 34.4% linoleic acid, 2.2% α-linolenic acid of PUFA, and 2.9 and 21.5% of stearic acid and palmitic acid of SFA, respectively^78^. The present results were also comparable with those reported by Resurreccion and Juliano^79^.

Similarly, the variations in the fatty acid profile of the present study proved better in having lower maximum limits for SFA and higher maximum limits for linoleic and linolenic acid than those reported by Goffman et al.^80^, who obtained 13.9–22.1% for palmitic, 1.5–2.7% for stearic, 35.9–49.2% for oleic, 27.3–41.0% for linoleic and 1.0–1.9% for linolenic acid in rice bran. Stearic acid and arachidic acid were present in trace amounts in all the studied aromatic rice cultivars. Comparatively, the fatty acid profile of *Local Joha* was better than that of the remaining cultivars, as it possessed a high level of linoleic and linolenic acid and low saturated fatty acid content. In general, the fatty acid profile of *Joha* rice cultivars qualifies for the extraction of quality bran oil for consumption.

Iron and zinc are crucial in numerous metabolic processes in the human body. Inadequacies of zinc and iron in the human diet are associated with growth retardation, physical and cognitive impairment, anaemia, loss of immunity, vulnerability to infection, abnormal pregnancy and neuropsychological disorders^81^. Although improved rice varieties and crop management practices contributed to a two-fold increase in rice production in the past few decades^82^, breeding for high-yielding, quality rice is crucial to meet energy needs and ensure nutritional health in developing countries^83^. Fe and Zn are essential micronutrients in cell development and gene expression^84,85^. Iron and zinc deficiency is a severe nutritional problem for humans and is particularly prevalent among children and pregnant women, especially in developing countries. As identified in the current study, *Joha* rice cultivars with very high iron and zinc contents in brown rice can help iron and zinc biofortification through conventional breeding or biotechnology-based approaches. Increasing the iron and zinc content and bioavailability in rice grains can benefit the human population, especially in developing countries. Substantial variations in brown rice's iron and zinc contents agreed with Chowdhury et al.^86^. A wide variation in iron and zinc contents of dehusked rice grains was evident among the Indian rice cultivars^87^. The range was between 5.1—441.5 µg/g (mean 67.8 µg/g) for iron and 2.12—39.4 µg/g (mean 23.8 µg/g) for zinc. The brown rice iron and zinc contents varied between 6.2—71.6 ppm and 26.2—67.3 ppm, respectively, in 126 rice accessions^88^. Vanlalsanga et al.^82^ also reported iron and zinc content in dehusked rice ranging from 11.42–215.62 ppm and 17.98–75.8 ppm, respectively in northeast Indian rice landraces. Brown rice has higher Zn and Fe contents than polished rice^89–91^. Therefore, emphasis should be given more to pre-breeding for increasing Zn and Fe contents in the polished rice, as the % loss during polishing depends on the degree and duration of polishing^92^ as well as location and variety^93^.The variation in protein content was in agreement with Banerjee et al.^94^, who reported 4.91 to 12.08% protein in 258 diverse rice landraces with a mean of 6.63 percent. Bajpai and Singh^45^ also noted low to medium amylose content. In aromatic rice, Semwal et al.^44^ also observed variation in aroma and accordingly classified the genotypes**.**

The number of alleles per locus (2.64) obtained in the present study was comparable with earlier reports by Shah et al.^95^, who reported 2.6, 2.75, and 2.3 alleles per locus, respectively. The mean allele number (2.64) obtained in the present study was higher than that of Meti et al.^96^, who detected 2.08 alleles per locus using 48 traditional indigenous aromatic rice germplasm grown under the eastern part of India through 12 polymorphic SSR loci. Prasad et al.^58^ obtained 82 alleles amplified by 27 polymorphic SSR markers, averaging 3.04 per locus in 208 aromatic rice genotypes of India. In contrast, the mean alleles (2.64) detected were markedly lower than the average number of alleles reported in previous diversity studies by Rahman et al.^97^, who obtained an average of 4.4 and 4.18 alleles per locus, respectively. The variability in the number of alleles detected per locus might be due to diverse genotypes and the selection of different SSR primers with scorable alleles. Similarly, Sajib et al.^98^ reported a significant allele frequency ranging from 0.41 to 0.91; Shah et al.^95^ noted a range of 0.425 to 0.975 with an average of 0.647, and Kumar et al.^99^ observed it to vary from 0.510 to 0.970, averaging 0.74. More alleles generated by SSR markers suggest this marker system's usefulness for detecting genetic polymorphisms. Aljumaili et al.^100^ detected 1.48 effective alleles per SSR locus among 53 rice cultivars.

In contrast, the effective allele number detected in the present study was lower than the average number of effective alleles (5.51) reported by Yelome et al.^101^ among West African rice accessions. Aljumaili et al.^100^ reported a similar Shannon's informative index by evaluating fifty-three aromatic rice accessions using 32 SSR markers, and they obtained a mean value of 0.580. The high value of Shannon's information index indicated the presence of high genetic diversity in the rice germplasm under consideration^102^. In contrast, Shah et al.^95^ recorded an average gene diversity of 0.448, ranging from 0.049 to 0.664, whereas Kumar et al.^99^ reported gene diversity ranging from 0.045 to 0.588 with a mean of 0.340. Similarly, the low level of observed heterozygosity, as also reported by Yelome et al.^101^, could be due to the autogamous mode of reproduction in rice. The ten highly informative markers detected in our study could be used to identify the twenty aromatic rice cultivars. The polymorphism detected in the present study was consistent with the reported mean PIC values in previous works^98^. However, Nadia et al.^103^ said an average PIC value of 0.84, markedly higher than the present average PIC value. Sufficient polymorphism by the 66 SSR markers among the twenty indigenous *Joha* rice cultivars justifies their proper classification and use in the genetic improvement programme based on the extent of genetic variation for desirable alleles. Our study identified 28 unique alleles specific to the 13 *Joha* rice cultivars. Shamim et al.^104^ detected 79 private alleles at 28 SSR loci in 16 locally adapted rice varieties and emphasized their importance in rice breeding. Prasad et al.^58^ considered a genotype-specific SSR locus amplifying a distinct band as unique or less frequent and detected four of 27 polymorphic markers amplified in 13 aromatic rice accessions. The unique SSR alleles represent a rich source of genetic diversity and diagnostic tools in aromatic rice breeding.

The Jaccard’s coefficients of similarity among the 20 *Joha* rice cultivars ranged from 0.24 between *Kon Joha-1* and *Manimuni Joha* to 0.78 between *Kon Joha-5* and *Joha-Golaghat*, with an average of 0.55, suggesting diverse nature of the genotypes under study. Similar to the present clustering pattern, Meti et al.^96^ obtained two major clusters for 48 aromatic rice genotypes from Odisha using 12 SSR markers at 49 per cent genetic similarity. Shah et al.^95^ effectively differentiated the basmati cultivars from non-basmati cultivars based on cluster analysis with 24 microsatellite loci, classifying 40 rice cultivars into three groups. Islam et al.^105^ used phylogenetic and model-based population structure analyses and classified 113 aromatic rice germplasm into three groups. Thus, SSR markers provided an adequate resolution to discriminate between aromatic rice accessions, and they could serve as a potential tool in identifying and characterizing genetically distant accessions from various sources. The microsatellite assays generated genotype-specific alleles in some of the cultivars evaluated for DNA fingerprints for cultivar identification and differentiation of aromatic rice. DNA fingerprints would be enormously helpful for establishing and defending proprietary rights and maintaining cultivar purity.

## Conclusion

Morpho-molecular and biochemical profiling of a panel of Assam’s popular indigenous *Joha* rice cultivars has been a step forward for exploiting variability in this unique rice class to improve its inherently low-yield potential through breeding. Our study revealed that the *Joha* rice cultivars are highly diverse in yield and quality traits. Recombination breeding among the trait-specific genotypes such as the early maturing *Kola Joha* with large grains and high biological yield, *Soru Joha (Tinsukia)* with high grain yield ha^−1^, short-statured *Keteki Joha* with high productive tillers, *Kon Joha-5* with more filled grains and *Joha (Bihpuria)* with high harvest index would provide a broad genetic base for aromatic *Joha* rice improvement programs. The low to high degree of dissimilarity among the accessions suggests the high molecular level diversity among the aromatic rice cultivars and their possible utilization in breeding programs to develop elite aromatic rice varieties. The unique alleles in 13 *Joha* cultivars are a rich source of genetic diversity to help marker-based identification/differentiation of aromatic rice cultivars and maintain this high-quality product's integrity to benefit farmers and consumers. The *Joha* rice cultivar *Soru Joha* (*Tinsukia*), with the highest yield (3012 kg ha^−1^), high spikelet fertility (90.9%), and high Fe content (61.09 mg kg^−1^), could serve as an immediate resource for mainstreaming. The *Joha* rice cultivar fatty acid profile qualified to extract quality bran oil for consumption. Our study opened the scope for value addition through nutritional profiling and yield enhancement through crossbreeding within this specialty rice class without compromising inherent quality characteristics. High-yielding nutri-rich *Joha* rice would encourage farmers’ adoption of wide-scale cultivation and increase farm income. At the same time, these valuable rice germplasm need to be collected, preserved, characterized, genetically enhanced, and documented in the context of intellectual property rights (IPR). Studying the medicinal properties of the *Joha* rice cultivars is another vital area of research.

## Methods

### Phenotypic characterization

The experiments were carried out during *the Sali* season of 2018 and 2019 at the Instructional-cum-Research (ICR) Farm, Assam Agricultural University. All molecular work, including DNA extraction, PCR, and gel electrophoresis, was performed in the Mutation Breeding Section-I Laboratory of Nuclear Agriculture and Biotechnology Division (NA&BTD), Bhabha Atomic Research Centre, Trombay. The field experimental site is located at 26°45^/^north latitude and 94°12^/^east longitude and has an elevation of 86.6 m above the mean sea level. The soils of the experimental site belong to the order Inceptisols with sandy loam texture and pH 4.8. The status of organic carbon, available nitrogen and phosphorus was medium, and available potassium was low. The growing situation was shallow land with a maximum water depth of 30 cm during the peak monsoon.

Twenty indigenous scented (*Joha*) rice cultivars collected from different agro-climatic zones of Assam (Table 1) were grown in a randomized complete block design with three replications. The seedlings' age was 30 days at transplanting in the main field. Each genotype constituted ten rows of 2.5 m long spaced 20 cm apart with one seedling per hill. A fertilizer dose of 60 kg N, 20 kg P_2_O_5_, and 40 kg K_2_O was applied as per the *Sali* rice recommendation for Assam. The standard agronomic practices recommended for the state of Assam were adopted in both experiments. Observations were recorded according to the National Test Guidelines for DUS test in rice developed by the Directorate of Rice Research, Hyderabad^15^. The yield-attributing traits were based on five random plants per replication, while days to flowering and maturity were recorded per plot. Additional data were recorded on flag leaf length, breadth and area, days to first flowering, plant height (cm), spikelet fertility (%),biological yield plant^−1^ (g), harvest index (%), grain yield plant^−1^ (g), grain yield ha^−1^ (kg), protein content (%), iron (Fe) and zinc (Zn) content (mg kg^−1^), fatty acid profile in rice bran.

### Estimation of total protein

Nitrogen was estimated in the samples of polished rice of the selected accessions by the modified Micro-Kjeldahl method^16^. The percentage of nitrogen was multiplied by the conversion factor of 5.95^17^ to estimate the total protein content. About 0.5 g of rice flour was digested at 400 °C in the presence of concentrated H_2_SO_4_ and a mixture of K_2_SO_4_ and CuSO_4_, followed by distillation using 4% boric acid and 40% NaOH solution. The distilled samples were titrated against the 0.1 N sulphuric acid until the first pink colour appeared at the last point. The titer value was used to calculate the per cent nitrogen.

$$Nitrogen \% = \frac{{14 \times \left( {Normality\;of\;acid} \right) \times Titrant\;value \left( {burette\;reading} \right)}}{Sample\;weight \left( g \right)} \times 100$$

$$Protein \% = Nitrogen \% \times 5.95$$

### Estimation of iron (Fe) and zinc (Zn)

The seeds harvested from the 20 selected cultivars were used for the zinc and iron estimation. The samples were accurately weighed (0.5 g each) and placed in a 250 ml digestion tube with nitric acid. To each sample, 5 ml of 65% HNO_3_ was added and boiled gently over a digester (90 °C) for 1–2 h. or until obtaining a clear solution. Subsequently, 2.5 ml of 65% HNO_3_ was added, and the tubes were further heated until total digestion^18^. During digestion, the tubes' inner walls were washed with 2 ml of deionized water to avoid loss of the samples. The samples were then filtered using Whatman No. 42 (2.5 μm particle retention) filter papers, and the final volume was made up to 25 ml by adding sufficient deionized water. Fe and Zn standard solutions were prepared in deionized water. The signal of the blank solution was recorded in duplicate. The signals of the standard solutions (in duplicate) were taken using the lamp corresponding to each element. The calibration curves for Fe and Zn were prepared after subtracting the blank from the recorded signals. The Fe/Zn solution was absorbed using the respective elements. The concentrations of Fe and Zn were calculated from the Fe and Zn calibration curves, respectively.

### Fatty acid profiling in rice bran

Fatty acid estimation of each rice cultivar was done in duplicates by Gas Chromatography technique (Shimadzu, Kyoto, Japan) at the Nuclear Agriculture and Biotechnology Division of Bhabha Atomic Research Centre, Trombay. After the hulling process, decorticated grains (brown rice) of all 20 rice cultivars were used for milling up to 5% (approx.) by using a McGill No. 2 miller (Rapsilver Supply Co. Inc., Brookshire, TX). After milling every sample, their bran was collected into small, stripped polythene and adequately labeled. The rice bran of each rice cultivar was stored at 4 °C to prevent the harmful activity of the lipase enzyme. About 200 mg of rice bran of each genotype was taken in a 50 ml glass test tube. One ml each of methanol (analytical grade) and 0.5 M sodium methoxide (analytical grade) was added to the tube. The tubes were shaken thoroughly by vortex and kept for 20 min at room temperature. Then, all the tubes were kept in a water bath at 500 °C for 1 h and then for 10 min at room temperature for cooling. Two ml each of HPLC-grade petroleum ether and deionized water were added to each tube, vortexed properly, and kept for one hour of phase separation. The supernatant was extracted from each tube using a 1 ml micro-pipette and taken in 1.8 ml clear GC vials for analyzing the samples by gas chromatography (GC SOLUTION, Shimadzu, Kyoto, Japan). The fatty acid concentration was recorded by normalizing peak areas using GC SOLUTION software (Shimadzu, Kyoto, Japan) and converted to a percentage. For further analysis, fatty acid proportional contents were arcsine transformed according to Sokal and Rohlf^19^.

### Genomic DNA extraction and purification

Cultivars' seeds were grown in a growth chamber, maintaining a temperature of 30 °C, 10 h of light, and 85% relative humidity. The leaves were harvested in liquid nitrogen for DNA isolation at the three-leaf stage,. The genomic DNA was isolated by the cetyl trimethyl ammonium bromide (CTAB) method^20^. The concentration and quality of genomic DNA were determined by measuring the absorbance at 260 and 280 nm. The samples showing a 260/280 ratio exceeding 1.8 were good-quality DNA free from protein contamination. The quality of the DNA fragment was also confirmed by 0.8% agarose gel electrophoresis using 1XTBE buffer at 100 V for 90 min.

### Primer selection and PCR amplification

Seventy-one SSR markers of genome-wide distribution (Supplementary Table S1), selected from various published literature and the Gramene database (www.gramene.org), were used for genotyping the cultivars.PCR was performed in a 25 μL mixture containing 1 μL (25 ng/μL) template DNA, 2.5 μL of 10x PCR buffer with 25 mM MgCl_2_, 1.0 μL 5 mM of each forward and reverse SSR primer, 1.0 μL 10 mM dNTPs and 0.2 μL *Taq* DNA polymerase. PCRs were performed in a thermal cycler (Eppendorf, Hamburg, Germany). The amplification profile consisted of initial denaturation for 2 min at 95 °C, 35–40 cycles of denaturation at 95 °C, annealing at 50–60 °C, and extension at 72 °C. After that, the final extension was carried out at 72 °C for 7 min. PCR products of SSR markers were resolved on a capillary electrophoresis system (Qiagen Pvt. Ltd., Hamburg, Germany). Only intense bands were scored based on their product size determined from a ladder of known molecular weights. The presence of a product in a particular genotype was designated as '1', and the absence as '0'.

### Research involving plants

The aromatic rice cultivars (*Oryza sativa* L.) used in this study are maintained in the Department of Plant Breeding and Genetics, Assam Agricultural University, Jorhat-13, Assam, India. This study complied with institutional and national guidelines for experimental research involving plants.

### Data analysis

A pooled ANOVA was performed for the traits over the two years, considering replication, genotype, and environment as fixed effects^21^ in MS Excel 2007. Genetic parameters were based on the formulae given by Burton^22^ for GCV and PCV, Hanson, Robinson, and Comstock^23^ for heritability, and Allard^24^ for expected genetic advances in MS Excel 2007. Mahalanobis *D*^*2*^ analysis^25^ was performed in Windostat version 9.2 (http://www.windostat.org). The principal component analysis was computed using the ‘FACTOEXTRA’ package of R statistical software^26^. Also, the biplot analysis was carried out using the ‘GGplot2’ package of R statistical software^27^. Usual Euclidian distances between the cultivars were determined from the standardized data matrix in DARwin version 6.0.021^28^ and represented through cluster analysis using the unweighted neighbour-joining (UNJ) method by feeding the distance matrix as input data. Genetic relatedness among the genotypes was computed by using Jaccard's coefficient of similarity^29^, and a dendrogram was constructed illustrating the genetic relationship among the rice genotypes using the UNJ method as proposed by Gascuel^30^, which uses a criterion of weighted average in DARwin 6.0.021^28^. The number of different alleles amplified per locus (*Na*), major allele frequency (*MAF*), number of effective alleles (*Ne*), Shannon's information index (*I*), observed heterozygosity (*Ho*), expected heterozygosity (*He*), and polymorphism information content (*PIC*) values were calculated using GenAlEx version 6.5^31^.

### Supplementary Information

Supplementary Information.

## Data Availability

The supplementary information file includes all data generated or analyzed during this investigation.
