# Supplementary material for: Morpho-molecular and nutritional profiling for yield improvement and value addition of indigenous aromatic Joha rice of Assam
Source: Sci Rep. 2024 Feb 12;14:3509. doi: 10.1038/s41598-023-42874-9 (PMC10861566; doi:10.1038/s41598-023-42874-9)
Supplement: Supplementary file 1 — Supplementary Information. [file 41598_2023_42874_MOESM1_ESM.pdf]

Manuscript Title: **Morpho-molecular and nutritional profiling for yield improvement and value addition of indigenous aromatic *Joha* rice of Assam**

Author information

Authors and Affiliations

1. **Krishi Vigyan Kendra, Karimganj, Assam Agricultural University, Karimganj-788712, Assam, India**

   Dibosh Bordoloi

   Email ID: dibosh.bordoloi@aau.ac.in

2. **Department of Plant Breeding and Genetics, Assam Agricultural University, Jorhat-785013, Assam, India**

   Debojit Sarma (Corresponding author)

   Email ID: debojit.sarma@aau.ac.in

   Nagendra Sarma Barua

   Email ID: nagendra.sarmabarua@aau.ac.in

3. **Department of Crop Physiology, Assam Agricultural University, Jorhat-785013, Assam, India**

   Ranjan Das

   Email ID: ranjan.das@aau.ac.in

4. **Nuclear Agriculture and Biotechnology Division, Bhabha Atomic Research Centre, Mumbai-400085, India**

   Bikram Kishore Das

   Email ID: bkdas@barc.gov.in

Supplementary Table S1: Details of SSR primers used in the study

Sl No	Designation	Forward primer	Reverse primer	Chr. No.	Expected size (bp)	Annealing Temp (°C)
1	RM495	AATCCAAGGTGCAGAGATGG	CAACGATGACGAACACAACC	1	159	56.2
2	RM283	GTCTACATGTACCCTTGTTGGG	CGGCATGAGAGTCTGTGATG	1	151	59.1
3	RM237	CAAATCCCGACTGCTGTCC	TGGGAAGAGAGCACTACAGC	1	130	55.3
4	RM431	TCCTGCGAACTGAAGAGTTG	AGAGCAAAACCCTGGTTCAC	1	251	54.1
5	RM259	TGGAGTTTGAGAGGAGGG	CTTGTTGCATGGTGCCATGT	1	162	55.8
6	RM212	CCACTTTCAGCTACTACCAG	CACCCATTTGTCTCTCATTATG	1	136	51.3
7	RM23	CATTGGAGTGGAGGCTGG	GTCAGGCTTCTGCCATTCTC	1	145	58.5
8	RM1003	GATTCTTCCTCCCCTTCGTG	TTCCTGTCAGAACAGGGAGC	1	128	48.5
9	R1M7	ATTCCTGGTTCTACATTACTTA	CGCCTCACTAGAATATCGGA	1	182	48.5
10	R1M30	AAGGGGCCCTAATTTATCTAG	TGTTTACTTTGTTCTTGGACTG	1	244	51.3
11	RM154	ACCCTCTCCGCCTCGCCTCCTC	CTCCTCCTCCTGCGACCGCTCC	2	183	50.4
12	RM452	CTGATCGAGAGCGTTAAGGG	GGGATCAAACCACGTTTCTG	2	209	54.9
13	RM29	CAGGGACCCACCTGTCATAC	AACGTTGGTCATATCGGTGG	2	250	51.3
14	RM221	ACATGTCAGCATGCCACATC	TGCAAGAATCTGACCCGG	2	192	59.1
15	RM6641	GGGTCTCGATTCTCAGTTGG	CAGAACCACTCATGCACACC	2	160	56.2
16	RM279	GCGGGAGAGGGATCTCCT	GGCTAGGAGTTAACCTCGCG	2	174	59.1
17	OSR13	CATTTGTGCGTCACGGAGTA	AGCCACAGCGCCCATCTCTC	3	115	57.2
18	RM338	CACAGGAGCAGGAGAAGAGC	GGCAAACCGATCACTCAGTC	3	183	56.2
19	RM514	AGATTGATCTCCCATTCCCC	CACGAGCATATTACTAGTGG	3	259	54.6
20	RM489	ACTTGAGACGATCGGACACC	TCACCCATGGATGTTGTCAG	3	271	55.7
21	RM55	CCGTCGCCGTAGTAGAGAAG	TCCCGGTTATTTTAAGGCG	3	226	54.9
22	RM3562	GCACTCGTCTTTCGCTTCTC	TTAGGATTCCACAAAGGGGG	3	150	55.3
23	RM60	AGTCCCATGTTCCACTTCCG	ATGGCTACTGCCTGTACTAC	3	165	54.1
24	RM124	ATCGTCTGCGTTGCGGCTGCTG	CATGGATCACCGAGCTCCCCCC	4	271	57.6
25	RM1388	TTCAATGAGGCAAAGGTAAG	ATTGTAGCTTGGACTAGGGG	4	236	50.4
26	RM471	ACGCACAAGCAGATGATGAG	GGGAGAAGACGAATGTTTGC	4	106	54.9
27	RM17467	CGCGCTTATATATGTACTCCGTACCC	CTTTGAAGACCGACTTTGGTTGC	4	397	57.2
28	RM317	CATACTTACCAGTTCACCGCC	CTGGAGAGTGTCAGCTAGTTGA	4	155	56.2
29	RM161	TGCAGATGAGAAGCGGCGCCTC	TGTGTCATCAGACGGCGCTCCG	5	187	54.7
30	RM178	TCGCGTGAAAGATAAGCGGCGC	GATCACCGTTCCCTCCGCCTGC	5	117	57.2

31	RM87	CCTCTCCGATACACCGTATG	GCGAAGGTACGAAAGGAAAG	5	151	54.6
32	RM3322	CTTCTCCACCCATGCCAC	CCTGCAACGAACACCCAC	5	121	57.6
33	RM133	TTGGATTGTTTTGCTGGCTCGC	GGAACACGGGGTCGGAAGCGAC	6	230	58.9
34	RM510	AACCGGATTAGTTTCTCGCC	TGAGGACGACGAGCAGATTC	6	122	57.2
35	RM585	CAGTCTTGCTCCGTTTGTTG	CTGTGACTGACTTGGTCATAGG	6	233	54.7
36	RM20236	GTGAACTTGACCGTCCGTCTACC	ACAGGACACCGGATAGAACAAGG	6	123	58.9
37	RM7434	GGAGGAAAGGTTGGAGAAGG	TTTCCCGTATTCCATGAGCC	6	143	55.9
38	RM2126	ACAGATTCAGGCCTTGTTTA	TCTTGCAGTTTACAGACGAA	6	172	51.5
39	RM253	TCCTTCAAGAGTGCAAAACC	GCATTGTCATGTCGAAGCC	6	141	53.6
40	RM217	ATCGCAGCAATGCCTCGT	GGGTGTGAACAAAGACAC	6	133	53.8
41	RM125	ATCAGCAGCCATGGCAGCGACC	AGGGGATCATGTGCCGAAGGCC	7	127	55.9
42	RM118	CCAATCGGAGCCACCGGAGAGC	CACATCCTCCAGCGACGCCGAG	7	156	51.5
43	RM481	TAGCTAGCCGATTGAATGGC	CTCCACCTCCTATGTTGTTG	7	169	53.1
44	RM11	TCTCCTCTTCCCCCGATC	ATAGCGGGCGAGGCTTAG	7	140	57.9
45	RM505	AGAGTTATGAGCCGGGTGTG	GATTTGGCGATCTTAGCAGC	7	199	55.1
46	RM501	GCCCAATTAATGTACAGGCG	ATATCGTTTAGCCGTGCTGC	7	179	54.8
47	RM152	GAAACCACCACACCTCACCG	CCGTAGACCTTCTTGAAGTAG	8	151	53.6
48	RM284	ATCTCTGATACTCCATCCATCC	CCTGTACGTTGATCCGAAGC	8	141	53.8
49	RM433	TGCGCTGAACTAAACACAGC	AGACAAACCTGGCCATTCAC	8	224	55.9
50	RM25	GGAAAGAATGATCTTTTCATGG	CTACCATCAAAACCAATGTTC	8	146	49.1
51	RM407	GATTGAGGAGACGAGCCATC	CTTTTTCAGATCTGCGCTCC	8	172	54.9
52	RM3481	CTCGTCGCGTTCGTCAAC	CATCTCATCACCTCACGTCG	8	224	55.8
53	RM3395	ACCTCATGTCCAGGTGGAAG	AGATTAGTGCCATGGCAAGG	8	97	55.7
54	RM316	CTAGTTGGGCATACGATGGC	ACGCTTATATGTTACGTCAAC	9	192	53.1
55	RM215	CAAAATGGAGCAGCAAGAGC	TGAGCACCTCCTTCTCTGTAG	9	148	57.9
56	RM105	GTCGTCGACCCATCGGAGCCAC	TGGTCGAGGTGGGGATCGGGTC	9	134	57.9
57	RM434	GCCTCATCCCTCTAACCCTC	CAAGAAAGATCAGTGCGTGG	9	143	55.0
58	RM271	TCAGATCTACAATTCCATCC	TCGGTGAGACCTAGAGAGCC	10	101	55.1
59	RM484	TCTCCCTCCTCACCATTGTC	TGCTGCCCTCTCTCTCTCTC	10	299	54.8
60	RM474	AAGATGTACGGGTGGCATTC	TATGAGCTGGTGAGCAATGG	10	252	58.5

61	RM171	AACGCGAGGACACGTACTTAC	ACGAGATACGTACGCCTTTG	10	328	57.3
62	RM228	CTGGCCATTAGTCCTTGG	GCTTGCGGCTCTGCTTAC	10	154	54.9
63	RM590	CATCTCCGCTCTCCATGC	GGAGTTGGGGTCTTGTTCG	10	137	57.2
64	RM591	CTAGCTAGCTGGCACCAGTG	TGGAGTCCGTGTTGTAGTCG	10	258	57.2
65	RM536	TCTCTCCTCTTGTTTGGCTC	ACACACCAACACGACCACAC	11	243	49.1
66	RM552	CGCAGTTGTGGATTTCAGTG	TGCTCAACGTTTGACTGTCC	11	195	48.5
67	RM229	CACTCACACGAACGACTGAC	CGCAGGTTCTTGTGAAATGT	11	116	57.3
68	RM26063	GATCCATATGCCTCTTCGATTGG	AACTCCAGCAGTGAGAGCGTAGC	11	121	57.9
69	RM21	ACAGTATTCCGTAGGCACGG	GCTCCATGAGGGTGGTAGAG	11	157	58.5
70	RM277	CGGTCAAATCATCACCTGAC	CAAGGCTTGCAAGGGAAG	12	124	54.9
71	RM27601	GCTTAGATGGTGCACAGGAAACC	GCTGTGTTTGTTTGAAGGTGTTGG	12	398	57.3

Supplementary Table S2: Twenty-two monomorphic characteristics of the 20 *Joha* rice cultivars

S. No.	Characteristics	States	Note	%
1	Coleoptile: colour	Green	2	100
2	Leaf: intensity of green colour	Medium	5	100
3	Leaf anthocyanin colouration	Present	9	100
4	Leaf sheath: anthocyanin coloration	Present	9	100
5	Leaf: auricles	Present	9	100
6	Leaf: anthocyanin colouration of auricles	Colourless	1	100
7	Leaf: collar	Present	9	100
8	Leaf: anthocyanin colouration of collar	Absent	1	100
9	Leaf: ligule	Present	9	100
10	Leaf: shape of ligule	Split	3	100
11	Leaf: colour of ligule	White	1	100
12	Leaf: width of blade	Narrow	3	100
13	Culm: attitude	Erect	1	100
14	Time of heading (50% of plants with panicles) (days)	Late	7	100
15	Male sterility	Absent	1	100
16	Panicle curvature of main axis	Dropping	7	100
17	Panicle: presence of secondary branch	Present	9	100
18	Panicle; exsertion	Well exerted	7	100
19	Decorticated grain: colour	Light brown	2	100
20	Endosperm presence of amylose	Present	9	100
21	Gelatinization temperature through alkali spreading value	Low	1	100
22	Decorticated grain: aroma	Present	9	100

Supplementary Table S3 : Usual Euclidean distances among the twenty *Joha* rice cultivars based on thirty-seven polymorphic morphological characteristics

Genotype	JOB	KAJ	ROJ	JOG	MMJ	KJM	KEJ	KJ1	SJT	KJ2	JEJ	KJ3	KJ4	KKJ	KJ5	LOJ	HAN	KJT	KOJ
KAJ	5.52																		
ROJ	8.69	6.76																	
JOG	6.79	7.24	8.96																
MMJ	6.08	6.00	6.87	6.97															
KJM	8.32	7.96	7.20	8.82	7.60														
KEJ	8.59	9.71	9.02	10.62	9.01	7.36													
KJ1	5.93	5.16	7.26	6.27	4.59	6.52	8.57												
SJT	5.47	6.91	8.73	8.14	5.85	8.44	8.46	5.88											
KJ2	8.25	6.72	7.29	8.71	6.68	7.57	10.07	6.94	7.72										
JEJ	8.13	7.08	6.57	8.70	7.17	4.61	7.84	7.06	7.95	7.27									
KJ3	10.18	10.2	9.42	7.65	8.70	8.56	10.65	9.54	9.23	8.39	8.90								
KJ4	6.51	4.49	7.20	7.78	6.05	7.62	9.02	5.38	6.52	7.26	6.90	10.10							
KKJ	9.01	8.56	9.19	9.27	7.73	8.97	10.06	6.83	8.86	9.16	8.94	10.90	8.65						
KJ5	9.40	9.08	8.34	9.75	8.22	5.36	7.47	7.97	9.08	7.46	5.69	8.22	8.78	9.12					
LOJ	11.53	12.39	11.56	11.65	11.51	10.2	7.69	11.52	10.53	12.59	10.17	10.47	11.30	12.35	9.88				
HAN	8.94	9.03	8.33	8.35	8.72	6.30	8.00	8.61	9.05	9.36	6.28	8.57	8.28	10.59	8.04	8.81			
KJT	10.24	9.85	9.66	8.57	9.56	9.95	11.03	8.39	10.24	10.74	10.32	10.86	9.86	6.22	10.95	12.03	9.29		
KOJ	5.88	7.54	8.36	8.68	7.56	9.36	9.57	7.14	6.21	9.49	9.75	10.51	8.60	10.40	10.26	11.78	10.25	10.63	
KOB	7.32	7.26	8.39	5.40	6.29	8.28	9.14	5.11	7.28	8.16	8.72	8.56	6.88	8.53	9.46	10.32	6.94	7.21	8.29

JOB: *Joha (Bihpuria)*; KAJ: *Kalijeera*; ROJ: *Ronga Joha*; JOG: *Joha (Golaghat)*; MMJ: *Manimuni Joha*; KJM: *Kon Joha (Moran)*; KEJ: *Keteki Joha*; KJ1: *Kon Joha-1*; SJT: *Soru Joha (Tinsukia)*; KJ2: *Kon Joha-2*; JEJ: *Jeera Joha*; KJ3: *Kon Joha-3*; KJ4: *Kon Joha-4*; KKJ: *Kunkuni Joha*; KJ5: *Kon Joha-5*; LOJ: *Local Joha*; HAN: *Harinarayan*; KJT: *Kon Joha (Teok)*; KOJ: *Kola Joha*; KOB: *Kon Joha (Bongaigaon)*

Supplementary Table S4: Pooled analysis of variance for the characters of the twenty indigenous *Joha* rice cultivars evaluated during the Sali season of 2018 and 2019

Source of variation	Rep/Year (4)	Year (1)	Cultivar (19)	Year x Cultivar (19)	Pooled Error (76)	Total (119)	CV (%)
Stem thickness (mm)	0.017	0.032	1.062**	0.003	0.014	0.180	2.930
Days to heading	1.117	13293.080**	116.359**	5.794**	0.810	131.765	0.790
Flag leaf length (cm)	22.428**	2.428**	113.840**	0.000	0.007	18.955	0.180
Flag leaf breath (cm)	0.003**	0.002**	0.005**	0.000	0.000	0.001	1.610
Flag leaf area (cm^2)	21.608**	4.575**	54.321**	0.153	0.210	9.597	1.620
Days to 50% flowering	2.917*	11800.830**	134.239**	12.833**	0.952	123.355	0.810
Days to maturity	7.929**	11800.830**	157.709**	12.833**	1.445	127.586	0.790
Plant height (cm)	62.921	15198.750**	643.155**	1.799	41.959	259.609	5.070
Productive tillers $Plant^{-1}$	11.556*	51.457**	15.345**	1.423	4.079	6.103	19.390
Panicle length (cm)	69.119**	41.961	20.917	17.693	16.974	19.681	15.760
Filled grains $Panicle^{-1}$	929.723	12808.370**	11184.250**	3211.616**	607.915	2825.632	13.910
Spikelet fertility (%)	5.026	2071.184**	111.783**	165.700**	10.628	68.665	3.750
1000-grain weights (g)	0.054	0.884*	70.231**	0.048	0.219	11.370	2.660
Grain length (mm)	0.023*	0.003	8.710**	0.000	0.007	1.396	1.140
Grain breadth (mm)	0.007**	0.001	0.329**	0.000	0.002	0.054	1.680
Grain length/breadth ratio	0.002	0.003	1.979**	0.000	0.004	0.319	2.120
Decorticated grain length (mm)	0.010*	0.003	4.657**	0.000	0.004	0.746	1.200
Decorticated grain breadth(mm)	0.001	0.001	0.212**	0.000	0.001	0.035	1.360
Decorticated grain length/breadth ratio	0.003	0.004	1.305**	0.000	0.002	0.210	1.870
Biological yield (g $Plant^{-1}$)	46.796	442.714**	78.453**	4.080	32.677	39.340	17.030
Grain yield (g $Plant^{-1}$)	27.370*	590.698**	17.850**	8.613	8.140	15.308	22.050
Harvest index (%)	101.778*	398.289**	79.442**	52.984	29.934	47.029	17.290
Grain yield (kg ha^{-1})	32108.325	438020.800**	1486216.000**	5898.026	13936.404	251897.300	5.870

*,**Significant at 5%and1% level, respectively; Figures within parentheses are degrees of freedom.

Supplementary Table S5: Mean performance of the twenty indigenous *Joha* rice cultivars evaluated during *Sali* season of 2018 and 2019

Cultivars	Stem thickness (mm)	Days to 1st flowering	Flag leaf length (cm)	Flag leaf breath (cm)	Flag leaf area (cm^2)	Days to 50% flowering	Days to maturity	Plant height (cm)	Productive tillers Plant^{-1}	Panicle length (cm)	Filled grains Panicle^{-1}
Joha (Bihpuria)	4.59^{b}	108.33^{a}	46.68^{j}	0.84^{a}	29.20^{e}	114.17^{b}	146.57^{c}	135.60^{ghi}	9.73^{def}	26.64	161.83^{def}
Kalijeera	4.27^{de}	109.67^{b}	45.03^{m}	0.77^{fg}	25.84^{i}	114.50^{bc}	146.50^{bc}	134.73^{fgh}	9.33^{def}	26.74	183.03^{bcd}
Ronga Joha	3.55^{j}	114.17^{ef}	54.41^{c}	0.83^{a}	34.09^{a}	119.17^{ef}	151.17^{e}	121.98^{c}	12.43^{abc}	26.67	112.43^{hi}
Joha (Golaghat)	4.42^{c}	108.50^{a}	53.29^{e}	0.77^{fg}	30.87^{d}	113.83^{b}	146.50^{bc}	132.50^{fg}	8.97^{ef}	25.18	188.20^{bcd}
Manimuni Joha	4.00^{fg}	110.50^{b}	49.51^{g}	0.79^{cde}	29.36^{e}	115.33^{c}	147.33^{c}	134.17^{fgh}	8.90^{ef}	25.72	228.90^{a}
Kon Joha (Moran)	3.96^{gh}	111.83^{c}	49.11^{h}	0.75^{h}	27.48^{g}	116.83^{d}	148.83^{d}	122.23^{c}	10.48^{cde}	27.07	232.83^{a}
Keteki Joha	3.00^{k}	119.33^{i}	43.13^{p}	0.81^{b}	26.22^{i}	127.50^{k}	162.03^{j}	98.90^{a}	13.80^{a}	27.28	145.23^{efg}
Kon Joha-1	3.84^{hi}	115.00^{f}	46.76^{j}	0.79^{cde}	27.48^{g}	120.00^{fg}	151.87^{ef}	129.70^{defg}	10.47^{cde}	24.63	190.67^{bc}
Soru Joha (Tinsukia)	4.28^{cde}	113.33^{de}	49.62^{f}	0.84^{a}	31.13^{d}	117.50^{d}	149.50^{d}	124.03^{cde}	10.23^{cde}	26.79	129.50^{ghi}
Kon Joha-2	4.90^{a}	120.83^{j}	46.76^{j}	0.75^{h}	26.10^{i}	125.33^{ij}	157.20^{h}	127.77^{cdef}	7.80^{f}	23.42	227.47^{a}
Jeera Joha	4.16^{e}	116.83^{g}	48.60^{i}	0.77^{fg}	28.10^{f}	122.00^{h}	153.87^{g}	140.70^{hi}	9.07^{ef}	26.52	234.27^{a}
Kon Joha-3	4.34^{cd}	116.67^{g}	44.19^{n}	0.79^{cde}	26.32^{i}	121.00^{gh}	152.87^{fg}	124.43^{cde}	10.53^{cde}	24.03	135.90^{fgh}
Kon Joha-4	4.19^{e}	120.50^{j}	45.43^{l}	0.76^{gh}	25.97^{i}	124.83^{i}	156.83^{h}	131.03^{efg}	9.39^{def}	26.72	189.90^{bcd}
Kunkuni Joha	4.00^{fg}	113.67^{de}	42.80^{q}	0.76^{gh}	24.53^{jk}	119.33^{ef}	151.73^{ef}	134.43^{fgh}	9.37^{def}	24.34	198.90^{b}
Kon Joha-5	4.62^{b}	118.00^{h}	40.84^{r}	0.79^{cde}	24.21^{k}	126.33^{j}	160.60^{i}	122.77^{cd}	8.77^{ef}	25.19	252.07^{a}
Local Joha	3.79^{i}	123.33^{k}	43.60^{o}	0.76^{gh}	24.75^{j}	128.50^{k}	161.17^{ij}	108.37^{b}	13.47^{ab}	26.11	138.27^{fgh}
Harinarayan	4.14^{ef}	112.67^{cd}	54.72^{b}	0.80^{bc}	32.92^{b}	118.67^{e}	151.87^{ef}	123.50^{cd}	11.47^{bcd}	32.25	168.60^{cde}
Kon Joha (Teok)	3.79^{i}	114.33^{ef}	54.82^{a}	0.78^{ef}	32.05^{c}	119.17^{ef}	151.17^{e}	135.17^{fgh}	11.57^{abcd}	24.56	154.33^{efg}
Kola Joha	3.98^{g}	108.00^{a}	53.80^{d}	0.80^{bc}	32.19^{c}	112.50^{a}	144.50^{a}	142.90^{i}	11.57^{abcd}	27.64	101.73^{i}
Kon Joha (Bongaigaon)	3.84^{hi}	114.67^{ef}	46.15^{k}	0.78^{ef}	26.93^{h}	119.50^{ef}	151.10^{e}	131.17^{efg}	11.03^{cde}	25.22	170.17^{cde}
**CD (5%)**	**0.1**	**0.73**	**0.07**	**0.01**	**0.37**	**0.79**	**0.98**	**5.27**	**1.64**	**-**	**20.05**

Cultivars	Spikelet fertility (%)	1000-grain weights (g)	Grain length (mm)	Grain breadth (mm)	Grain length/breadth ratio	Decorticated grain length (mm)	Decorticated grain breadth (mm)	Decorticated grain length/breadth ratio	Biological yield (g $Plant^{-1}$)	Grain yield (g $Plant^{-1}$)	Harvest index (%)	Grain yield (kg ha^{-1})
Joha (Bihpuria)	89.90^{abcde}	18.22^{de}	8.12^{e}	2.29^{gh}	3.54^{d}	6.15^{c}	2.16^{h}	2.85^{c}	34.31^{bc}	14.83^{ab}	36.40^{a}	2621.67^{b}
Kalijeera	88.41^{cdef}	15.36^{j}	6.33^{k}	2.40^{e}	2.64^{i}	4.48^{j}	2.24^{ef}	2.01^{hi}	28.98^{c}	13.00^{bc}	35.80^{ab}	1986.67^{de}
Ronga Joha	85.67^{fgh}	21.64^{c}	7.98^{f}	2.59^{d}	3.08^{f}	5.76^{e}	2.48^{b}	2.32^{f}	40.01^{a}	17.20^{a}	35.21^{ab}	2030.00^{d}
Joha (Golaghat)	90.61^{abcd}	18.64^{d}	7.65^{h}	2.43^{e}	3.14^{f}	5.26^{g}	2.24^{e}	2.35^{f}	33.37^{c}	13.02^{bc}	34.05^{abc}	1843.33^{f}
Manimuni Joha	88.79^{bcdef}	14.57^{k}	6.27^{kl}	2.57^{d}	2.43^{j}	4.74^{h}	2.43^{c}	1.95^{i}	33.83^{bc}	13.00^{bc}	31.86^{abcd}	1831.67^{f}
Kon Joha (Moran)	87.43^{cdef}	13.78^{l}	6.16^{mn}	2.28^{gh}	2.70^{hi}	4.23^{lm}	2.17^{gh}	1.96^{i}	30.50^{c}	12.03^{bc}	32.75^{abcd}	2593.33^{b}
Keteki Joha	86.25^{efg}	17.17^{f}	8.24^{d}	2.09^{j}	3.96^{b}	6.46^{b}	2.02^{l}	3.20^{b}	31.36^{c}	14.55^{ab}	35.15^{ab}	1668.33^{g}
Kon Joha-1	85.93^{fg}	15.94^{hi}	6.32^{k}	2.33^{fg}	2.72^{hi}	4.49^{j}	2.06^{jkl}	2.18^{g}	35.51^{bc}	13.39^{bc}	30.63^{abcde}	1878.33^{ef}
Soru Joha (Tinsukia)	90.92^{abc}	22.50^{b}	9.04^{b}	2.42^{e}	3.74^{c}	6.97^{a}	2.10^{i}	3.32^{a}	31.97^{c}	12.57^{bc}	33.89^{abc}	3011.67^{a}
Kon Joha-2	82.10^{hij}	18.42^{d}	6.15^{mn}	2.79^{b}	2.20^{k}	4.76^{h}	2.57^{a}	1.85^{j}	31.39^{c}	8.64^{d}	22.99^{f}	1000.00^{i}
Jeera Joha	93.03^{a}	14.69^{k}	6.47^{j}	2.38^{ef}	2.72^{hi}	4.34^{k}	2.03^{kl}	2.14^{g}	32.57^{c}	11.66^{bcd}	29.98^{bcde}	1801.67^{fg}
Kon Joha-3	83.36^{ghi}	22.06^{bc}	9.30^{a}	2.72^{c}	3.43^{e}	5.97^{d}	2.26^{e}	2.64^{d}	34.67^{bc}	13.37^{bc}	30.15^{abcde}	1803.33^{fg}
Kon Joha-4	86.12^{fg}	14.60^{k}	6.11^{mn}	2.15^{i}	2.85^{g}	4.59^{i}	1.89^{m}	2.43^{e}	30.68^{c}	12.59^{bc}	34.44^{abc}	2553.33^{b}
Kunkuni Joha	87.03^{defg}	13.02^{m}	6.08^{n}	2.19^{i}	2.78^{gh}	4.29^{kl}	2.09^{ij}	2.05^{h}	32.71^{c}	11.74^{bcd}	28.51^{cdef}	2378.33^{c}
Kon Joha-5	79.89^{ij}	16.31^{gh}	5.95^{o}	2.87^{a}	2.08^{l}	4.17^{mn}	2.33^{d}	1.79^{k}	34.96^{bc}	11.65^{bcd}	28.70^{cdef}	1853.33^{ef}
Local Joha	80.47^{ij}	18.36^{de}	9.26^{a}	2.15^{i}	4.30^{a}	6.10^{c}	1.92^{m}	3.18^{b}	33.86^{bc}	14.45^{ab}	33.59^{abcd}	1675.00^{g}
Harinarayan	78.97^{j}	16.59^{g}	6.20^{lm}	2.25^{h}	2.75^{h}	4.13^{n}	2.20^{fgh}	1.88^{j}	33.97^{bc}	11.10^{cd}	25.31^{ef}	1468.33^{h}
Kon Joha (Teok)	92.61^{a}	15.65^{ij}	7.76^{g}	2.19^{i}	3.54^{d}	5.47^{f}	2.06^{jk}	2.65^{d}	31.31^{c}	13.04^{bc}	31.97^{abcd}	2286.67^{c}
Kola Joha	92.29^{ab}	26.69^{a}	8.76^{c}	2.78^{b}	3.16^{f}	6.11^{c}	2.48^{b}	2.46^{e}	44.97a	14.26^{abc}	27.52^{def}	1366.67^{h}
Kon Joha (Bongaigaon)	91.09^{abc}	17.87^{e}	7.45^{i}	2.41^{e}	3.10^{f}	5.20^{g}	2.20^{fg}	2.36^{f}	30.21^{c}	12.68^{bc}	34.15^{abc}	2590.00^{b}
**CD (5%)**	**2.65**	**0.38**	**0.07**	**0.03**	**0.05**	**0.05**	**0.02**	**0.04**	**4.65**	**2.32**	**4.45**	**95.99**

Supplementary Table S6: WILKS TEST and ANOVA for dispersion

WILKS TEST					
Determinant of Error Matrix			1.57E+01		
Determinant of Error + Variety Matrix			0.00E+00		
Wilk's Criterion					
M	91.5 V statistics	0			
Degree of Freedom	437 Probability	1			
ANOVAforDISPERSION					
Source of Variations	df	Sum of Squares	Mean Squares	F Ratio	Probability
Varieties	19	-1.57E+01	-8.25E-01	-4.95E+00	0.00000***
Error	94	1.57E+01	1.67E-01		
Total	113	0.00E+00	0.00E+00		

Supplementary Table S7: Eigenvalue, contribution of variability and factor loadings for the principal component axes

Parameter	Principle Component (PC)				
	1	2	3	4	5
Eigenvalue	7.31	5.57	2.91	2.00	1.09
Variability (%)	33.23	25.34	13.23	9.11	4.95
Cumulative variability (%)	33.23	58.57	71.81	80.91	85.87
Traits	Factor loadings after Varimax rotation				
Stem thickness (mm)	0.55	-0.34	0.29	-0.39	-0.33
Days to heading	0.16	0.84	0.35	0.02	-0.13
Flag leaf length (cm)	-0.33	-0.67	-0.11	0.41	-0.31
Flag leaf breath (cm)	-0.66	-0.26	0.15	0.09	0.16
Flag leaf area (cm^2)	-0.50	-0.65	-0.04	0.39	-0.21
Days to 50% flowering	0.17	0.88	0.35	0.08	-0.04
Days to maturity	0.15	0.88	0.36	0.13	-0.01
Plant height (cm)	0.30	-0.76	-0.23	-0.27	-0.11
Productive tillers $Plant^{-1}$	-0.76	0.39	0.02	0.40	0.08
Panicle length (cm)	-0.26	-0.12	-0.16	0.75	-0.19
Filled grains $Panicle^{-1}$	0.91	0.06	-0.16	-0.03	0.10
Spikelet fertility (%)	-0.27	-0.46	-0.55	-0.41	-0.06
1000-grain weights (g)	-0.65	-0.34	0.60	-0.16	-0.13
Grain length (mm)	-0.87	0.08	0.25	-0.31	-0.16
Grain breadth (mm)	0.22	-0.49	0.76	-0.22	0.18
Grain length/breadth ratio	-0.85	0.38	-0.12	-0.16	-0.22
Decorticated grain length (mm)	-0.88	0.08	0.21	-0.34	-0.13
Decorticated grain breadth (mm)	0.14	-0.64	0.59	0.02	0.23
Decorticated grain length/breadth ratio	-0.82	0.39	-0.05	-0.31	-0.22
Biological yield (g $Plant^{-1}$)	-0.41	-0.47	0.52	0.21	0.23
Harvest index (%)	-0.51	0.17	-0.56	-0.28	0.40
Grain yield (g $Plant^{-1}$)	-0.80	-0.02	-0.09	0.03	0.53

Supplementary Table S8: Contribution of the cultivars to the first two principal component axes

Cultivars	PC1	PC2
Joha (Bihpuria)	0.660	0.916
Kalijeera	-0.730	0.299
Ronga Joha	1.219	1.109
Joha (Golaghat)	-0.195	1.056
Manimuni Joha	-0.891	0.812
Kon Joha (Moran)	-0.766	-0.157
Keteki Joha	1.912	-1.771
Kon Joha-1	-0.314	-0.232
Soru Joha (Tinsukia)	1.273	0.582
Kon Joha-2	-1.817	-0.543
Jeera Joha	-0.841	-0.185
Kon Joha-3	0.542	-0.119
Kon Joha-4	-0.335	-1.174
Kunkuni Joha	-0.892	-0.555
Kon Joha-5	-1.388	-0.915
Local Joha	1.636	-1.985
Harinarayan	-0.393	0.374
Kon Joha (Teok)	0.516	0.260
Kola Joha	0.717	2.343
Kon Joha (Bongaigaon)	0.088	-0.115

Supplementary Table S9: Scoring of the twenty indigenous aromatic rice cultivars with seventy-one SSR markers

Marker Name	Allele (bp)	*Joha (Bihpuria)*	*Kalijeera*	*Ronga Joha*	*Joha (Golaghat)*	*Manimuni Joha*	*Kon Joha (Moran)*	*Keteki Joha*	*Kon Joha-1*	*Soru Joha (Tinsukia)*	*Kon Joha-2*	*Jeera Joha*	*Kon Joha -3*	*Kon Joha-4*	*Kunkuni Joha*	*Kon Joha-5*	*Local Joha*	*Harinarayan*	*Kon Joha (Teok)*	*Kola Joha*	*Kon Joha (Bongaigaon)*
RM495	160	1	1	1	1	1	1	1	1	1	1	0	1	0	1	1	1	1	1	1	1
RM495	149	0	0	0	0	0	0	0	0	0	0	1	0	1	0	0	0	0	0	0	0
RM 283	185	0	1	0	0	1	1	0	1	0	1	0	0	0	0	0	1	0	1	1	0
RM 283	175	0	0	1	0	0	0	0	0	1	0	0	0	0	0	0	0	0	0	0	1
RM 283	165	0	0	0	0	0	0	0	0	0	0	0	1	0	1	1	0	1	0	0	0
RM 283	155	1	0	0	1	0	0	1	0	0	0	1	0	1	0	0	0	0	0	0	0
RM 237	141	0	0	0	0	0	0	0	0	0	0	0	1	0	0	0	0	0	0	0	0
RM 237	134	1	1	1	1	1	1	1	1	1	1	1	0	1	1	1	1	1	1	1	1
RM431	260	0	0	0	0	0	0	0	0	0	0	0	0	0	0	1	0	0	0	0	0
RM431	252	0	0	0	1	0	1	1	0	0	0	0	1	0	0	0	0	1	0	0	0
RM431	242	1	1	1	0	1	0	0	1	1	1	1	0	1	1	0	1	0	1	1	1
RM154	200	0	0	0	0	0	0	0	0	0	0	0	0	0	0	0	0	0	0	1	0
RM154	188	1	1	1	1	1	1	1	1	1	0	0	0	0	1	0	1	1	1	0	1
RM154	170	0	0	0	0	0	0	0	0	0	1	1	1	1	0	1	0	0	0	0	0
OSR 13	95	0	1	0	0	0	0	0	0	0	1	0	0	0	0	0	1	0	0	0	0
OSR 13	105	1	0	1	1	1	1	1	1	1	0	1	1	1	1	0	0	1	1	1	1
OSR 13	117	0	0	0	0	0	0	0	0	0	0	0	0	0	0	1	0	0	0	0	0
RM338	191	0	1	0	0	0	0	1	0	0	1	1	0	1	0	1	0	0	0	0	1
RM338	183	1	0	1	1	1	1	0	1	1	0	0	1	0	1	0	1	1	1	1	0
RM514	269	0	0	0	0	1	0	0	1	0	1	0	0	0	1	1	0	0	1	0	1
RM514	245	0	1	1	1	0	1	1	0	1	0	1	1	1	0	0	1	1	0	1	0
RM514	179	1	0	0	0	0	0	0	0	0	0	0	0	0	0	0	0	0	0	0	0

RM124	272	0	0	1	0	0	0	1	0	0	0	0	0	0	0	0	0	0	0	0	0
RM124	266	1	1	0	1	1	1	0	1	1	1	1	1	1	1	1	1	1	1	1	1
RM 161	123	1	0	1	0	0	0	0	1	1	0	0	1	0	0	1	0	1	1	1	1
RM 161	113	0	1	0	1	1	1	1	0	0	1	1	0	1	1	0	1	0	0	0	0
RM133	264	0	0	0	1	1	0	0	0	0	0	0	0	0	0	0	0	0	0	0	0
RM133	232	1	1	1	0	0	1	1	1	1	1	0	0	0	1	1	1	1	1	1	1
RM133	Null	0	0	0	0	0	0	0	0	0	0	1	1	1	0	0	0	0	0	0	0
RM 125	175	0	0	0	0	0	0	0	0	0	0	0	0	1	0	0	0	0	0	0	0
RM 125	140	0	0	0	0	0	0	0	0	0	0	1	1	0	0	0	0	0	0	0	0
RM 125	128	1	1	1	1	1	1	1	1	1	1	0	0	0	1	1	1	1	1	1	1
RM 118	362	0	0	0	0	0	0	0	0	0	0	0	0	1	0	0	0	0	0	0	0
RM 118	356	1	0	0	0	0	0	0	0	0	1	1	1	0	1	1	0	0	0	0	0
RM 118	346	0	1	1	1	1	1	1	1	1	0	0	0	0	0	0	0	1	0	1	1
RM 118	336	0	0	0	0	0	0	0	0	0	0	0	0	0	0	0	1	0	1	0	0
RM 152	161	0	0	0	0	0	0	0	0	0	1	1	0	0	0	1	0	0	0	0	0
RM 152	151	1	1	1	1	1	1	1	1	1	0	0	1	1	1	0	1	1	1	1	1
RM284	152	1	1	1	0	1	0	1	1	1	1	1	1	0	1	1	1	1	1	1	1
RM284	142	0	0	0	0	0	1	0	0	0	0	0	0	1	0	0	0	0	0	0	0
RM284	null	0	0	0	1	0	0	0	0	0	0	0	0	0	0	0	0	0	0	0	0
RM316	220	0	0	0	1	1	1	0	0	1	0	0	0	0	0	0	0	0	0	0	1
RM316	212	0	1	1	0	0	0	0	0	0	0	0	0	0	1	1	0	1	0	1	0
RM316	203	0	0	0	0	0	0	1	0	0	1	0	0	0	0	0	1	0	1	0	0
RM316	Null	1	0	0	0	0	0	0	1	0	0	1	1	1	0	0	0	0	0	0	0
RM 215	153	1	1	1	1	1	0	1	1	1	1	1	1	1	1	1	1	1	1	1	1
RM 215	97	0	0	0	0	0	1	0	0	0	0	0	0	0	0	0	0	0	0	0	0
RM 271	108	0	0	0	0	0	0	0	0	0	0	0	0	0	0	1	1	0	0	0	0
RM 271	97	1	1	1	1	1	1	1	1	1	1	1	1	1	1	0	0	1	1	1	1
RM484	297	1	1	1	1	1	1	1	1	1	1	0	0	0	1	1	1	1	1	1	1
RM484	null	0	0	0	0	0	0	0	0	0	0	1	1	1	0	0	0	0	0	0	0

RM 536	236	1	0	0	1	1	1	1	1	1	1	1	1	0	0	0	0	0	0	0	0
RM 536	223	0	1	1	0	0	0	0	0	0	0	0	0	1	1	1	1	1	1	1	1
RM 277	140	1	1	0	1	0	0	1	0	0	0	0	1	0	1	0	0	1	1	0	0
RM 277	124	0	0	1	0	1	1	0	1	1	1	1	0	1	0	1	1	0	0	1	1
RM 259	185	0	1	0	0	1	1	0	1	1	1	0	0	0	0	0	1	0	1	1	0
RM 259	174	0	0	1	0	0	0	0	0	0	0	0	0	0	0	0	0	0	0	0	1
RM 259	160	1	0	0	1	0	0	1	0	0	0	1	1	1	1	1	0	1	0	0	0
RM489	265	1	1	1	0	1	1	0	1	1	0	0	1	0	1	1	0	1	1	1	1
RM489	255	0	0	0	1	0	0	1	0	0	1	1	0	0	0	0	1	0	0	0	0
RM489	160	0	0	0	0	0	0	0	0	0	0	0	0	1	0	0	0	0	0	0	0
RM55	330	0	0	0	0	0	0	0	0	0	0	0	1	1	0	0	0	0	0	0	0
RM55	245	0	1	0	0	0	0	0	0	0	0	0	0	0	0	0	0	0	0	0	0
RM55	235	1	0	1	1	1	1	1	1	1	1	1	0	0	1	1	1	1	1	1	1
RM 510	125	1	0	1	0	1	0	0	1	1	0	0	1	0	0	1	0	1	1	1	1
RM 510	115	0	1	0	1	0	1	1	0	0	1	1	0	1	1	0	1	0	0	0	0
RM474	255	0	1	1	1	1	1	0	1	1	0	0	0	0	1	0	1	0	1	1	1
RM474	228	0	0	0	0	0	0	0	0	0	1	1	0	1	0	1	0	1	0	0	0
RM474	Null	1	0	0	0	0	0	1	0	0	0	0	1	0	0	0	0	0	0	0	0
RM 171	358	1	0	0	0	0	0	1	0	0	1	1	1	1	0	1	0	0	0	0	0
RM 171	348	0	1	0	1	1	1	0	1	0	0	0	0	0	1	0	0	1	0	1	1
RM 171	338	0	0	1	0	0	0	0	0	1	0	0	0	0	0	0	1	0	1	0	0
RM212	120	1	1	1	1	1	1	1	1	1	1	0	0	1	1	1	1	1	1	1	1
RM212	null	0	0	0	0	0	0	0	0	0	0	1	1	0	0	0	0	0	0	0	0
RM23	162	0	0	0	0	1	1	1	1	0	1	0	1	1	1	1	1	0	0	1	1
RM23	147	1	1	1	1	0	0	0	0	1	0	0	0	0	0	0	0	1	1	0	0
RM23	Null	0	0	0	0	0	0	0	0	0	0	1	0	0	0	0	0	0	0	0	0
RM 229	142	1	0	1	1	1	0	1	0	1	0	1	1	1	1	0	1	1	1	1	1
RM 229	136	0	1	0	0	0	1	0	1	0	1	0	0	0	0	1	0	0	0	0	0

RM1003	145	1	0	0	0	0	0	1	1	0	0	0	0	1	1	0	1	0	0	0	0
RM1003	127	0	1	1	1	1	1	0	0	1	1	1	1	0	0	1	0	1	1	1	1
RM29	339	0	0	1	1	0	1	0	0	0	0	0	0	0	0	1	0	1	0	0	0
RM29	320	0	0	0	0	0	0	0	0	0	0	0	0	0	0	0	1	0	1	0	0
RM29	249	1	1	0	0	1	0	1	1	1	0	0	1	1	1	0	0	0	0	1	1
RM29	190	0	0	0	0	0	0	0	0	0	1	0	0	0	0	0	0	0	0	0	0
RM 221	214	0	0	0	0	0	0	0	0	0	1	0	0	0	0	1	1	1	0	0	0
RM 221	204	1	1	1	1	1	1	1	1	1	0	1	1	1	1	0	0	0	1	1	1
RM6641	183	0	0	0	0	0	0	0	0	0	0	1	0	0	0	0	0	0	0	0	0
RM6641	146	1	1	1	1	1	1	1	1	1	1	0	1	1	1	1	1	1	1	1	1
RM 279	168	0	0	0	0	0	0	0	0	0	0	0	0	0	0	0	0	0	0	0	1
RM 279	155	1	1	1	1	1	1	1	1	1	1	1	1	1	1	1	1	1	1	1	0
RM3562	150	0	0	0	0	0	0	0	1	0	1	0	1	0	0	0	0	0	0	0	1
RM3562	142	1	1	1	0	1	1	1	0	1	0	0	0	0	0	0	0	0	1	1	0
RM3562	Null	0	0	0	1	0	0	0	0	0	0	1	0	1	1	1	1	1	0	0	0
RM60	179	0	1	1	1	1	1	0	1	1	1	0	0	0	1	0	1	1	1	0	1
RM60	Null	1	0	0	0	0	0	1	0	0	0	1	1	1	0	1	0	0	0	1	0
RM1388	230	0	1	0	0	0	0	0	1	0	1	0	0	0	0	1	0	0	0	1	1
RM1388	217	1	0	0	1	0	1	1	0	1	0	1	1	1	1	0	0	0	0	0	0
RM1388	200	0	0	1	0	1	0	0	0	0	0	0	0	0	0	0	1	1	1	0	0
RM471	130	0	0	0	0	0	0	0	0	0	0	1	0	0	1	0	1	0	0	0	0
RM471	110	1	1	1	1	1	1	1	1	1	1	0	1	1	0	1	0	1	1	1	1
RM17467	420	0	0	1	1	0	1	0	0	0	1	0	0	0	0	0	0	0	0	0	0
RM17467	350	0	0	0	0	1	0	0	0	0	0	1	0	0	0	0	0	0	0	0	0
RM17467	390	1	1	0	0	0	0	1	1	1	0	0	1	1	1	1	1	1	1	1	1
RM 317	168	1	1	1	1	1	1	0	1	1	1	0	0	1	1	1	1	1	1	1	1
RM 317	Null	0	0	0	0	0	0	1	0	0	0	1	1	0	0	0	0	0	0	0	0
RM 187	168	1	1	1	0	1	1	0	1	1	1	0	1	1	1	1	1	1	1	1	1
RM 187	160	0	0	0	1	0	0	1	0	0	0	1	0	0	0	0	0	0	0	0	0

RM 3322	125	1	1	1	1	1	1	1	1	1	1	1	1	0	1	0	1	1	1	1	1
RM 3322	113	0	0	0	0	0	0	0	0	0	0	0	0	1	0	1	0	0	0	0	0
RM 585	213	0	1	0	0	0	1	0	0	0	0	0	0	1	0	0	0	0	0	0	0
RM 585	202	1	0	1	1	1	0	0	1	1	0	0	0	0	0	0	0	0	0	0	0
RM 585	192	0	0	0	0	0	0	1	0	0	1	1	1	0	1	1	1	0	1	1	1
RM 585	184	0	0	0	0	0	0	0	0	0	0	0	0	0	0	0	0	1	0	0	0
RM 20236	199	0	0	0	0	0	1	0	0	0	1	0	0	0	0	0	0	0	0	0	0
RM 20236	136	1	1	1	1	1	1	1	1	1	1	1	1	1	1	1	1	1	1	1	1
RM 7434	150	1	1	1	1	1	1	0	1	1	1	1	1	1	1	0	0	1	1	0	1
RM 7434	139	0	0	0	0	0	0	0	0	0	0	0	0	0	0	1	0	0	0	0	0
RM 7434	127	0	0	0	0	0	0	1	0	0	1	0	0	0	0	0	1	0	0	1	0
RM2126	155	1	1	1	1	1	0	0	1	1	1	1	1	1	1	1	1	1	1	1	1
RM2126	135	0	0	0	0	0	1	1	0	0	0	0	0	0	0	0	0	0	0	0	0
RM 253	164	1	1	0	0	0	1	0	1	1	1	1	1	1	0	0	0	0	0	0	1
RM 253	156	0	0	0	1	1	0	1	0	0	0	0	0	0	1	0	1	1	1	1	0
RM 253	131	0	0	0	0	0	0	0	0	0	0	0	0	0	0	1	0	0	0	0	0
RM217	160	0	0	0	0	0	0	0	0	0	0	1	0	0	0	0	0	0	0	1	0
RM217	120	1	1	1	1	1	1	1	1	1	1	0	1	1	1	1	1	1	1	0	1
RM434	147	1	1	1	1	1	1	0	1	1	1	1	1	1	1	0	0	1	1	1	1
RM434	138	0	0	0	0	0	0	0	0	0	0	0	0	0	0	1	0	0	0	0	0
RM434	126	0	0	0	0	0	0	1	0	0	0	0	0	0	0	0	1	0	0	0	0
RM 481	187	0	0	0	0	0	0	0	0	0	1	0	0	0	0	0	0	1	1	0	1
RM 481	159	1	1	1	1	1	1	1	1	1	0	1	1	0	1	0	1	0	0	1	0
RM 481	145	0	0	0	0	0	0	0	0	0	0	0	0	1	0	0	0	0	0	0	0
RM11	160	0	0	0	0	0	0	0	0	0	1	0	1	0	0	0	1	0	0	0	0
RM11	140	1	0	1	1	0	0	1	0	1	0	1	0	1	0	1	0	1	0	1	1
RM11	125	0	1	0	0	1	1	0	1	0	0	0	0	0	1	0	0	0	1	0	0

RM 505	198	1	0	0	0	0	0	0	0	0	0	0	0	0	0	0	0	0	0	0	0
RM 505	193	0	1	0	1	1	0	1	1	0	0	1	1	1	1	1	1	1	0	0	0
RM 505	183	0	0	1	0	0	0	0	0	1	1	0	0	0	0	0	0	0	1	1	1
RM501	182	1	1	0	0	0	1	0	0	0	0	0	0	0	0	1	0	0	0	0	0
RM501	165	0	0	0	1	1	0	1	1	1	1	1	1	1	1	0	1	1	1	1	1
RM501	155	0	0	1	0	0	0	0	0	0	0	0	0	0	0	0	0	0	0	0	0
RM25	160	0	0	1	0	0	0	0	0	0	0	1	0	0	0	0	0	0	0	0	0
RM25	142	1	1	0	1	1	1	1	1	1	1	0	1	1	1	0	1	1	1	1	1
RM25	150	0	0	0	0	0	0	0	0	0	0	0	0	0	0	1	0	0	0	0	0
RM407	173	0	0	0	0	0	0	0	0	1	1	0	0	0	0	1	0	0	1	0	0
RM407	163	1	1	1	0	1	1	1	1	0	0	0	1	1	1	0	1	1	0	1	1
RM407	155	0	0	0	1	0	0	0	0	0	0	1	0	0	0	0	0	0	0	0	0
RM3481	200	0	0	0	0	0	0	0	0	0	0	0	0	0	0	1	0	0	0	0	0
RM3481	193	1	1	1	1	1	1	1	1	1	1	1	1	1	1	0	1	1	1	1	1
RM3395	130	0	0	0	0	0	0	0	0	0	0	0	0	0	0	1	0	0	0	0	0
RM3395	122	0	0	0	0	0	0	1	0	0	0	0	0	0	0	0	0	0	0	0	0
RM3395	103	1	1	1	1	1	1	0	1	1	1	1	1	1	1	0	1	1	1	1	1
RM228	158	0	0	0	0	0	0	0	0	0	1	0	0	0	0	0	0	0	0	0	0
RM228	128	1	1	1	1	1	1	1	1	1	0	0	0	1	1	1	1	1	1	1	1
RM228	Null	0	0	0	0	0	0	0	0	0	0	1	1	0	0	0	0	0	0	0	0
RM590	150	0	0	0	1	0	0	0	0	0	1	1	1	0	0	0	0	0	0	0	0
RM590	142	1	1	1	0	1	1	1	1	1	0	0	0	1	1	1	1	1	1	1	1
RM591	292	0	0	0	0	0	0	1	0	1	0	0	0	0	0	0	0	0	1	0	1
RM591	272	1	1	1	0	1	1	0	1	0	0	1	1	1	1	1	1	1	0	1	0
RM591	262	0	0	0	1	0	0	0	0	0	1	0	0	0	0	0	0	0	0	0	0
RM 26063	160	1	0	0	0	0	0	0	0	0	0	0	0	1	0	0	0	0	0	0	0
RM 26063	152	0	1	0	1	1	0	0	1	1	1	1	0	0	1	1	0	0	1	1	1
RM 26063	135	0	0	0	0	0	1	1	0	0	0	0	1	0	0	0	0	1	0	0	0
RM 26063	120	0	0	1	0	0	0	0	0	0	0	0	0	0	0	0	1	0	0	0	0

RM21	174	0	0	0	0	0	0	0	0	0	1	0	0	0	0	0	0	0	0	0	0
RM21	134	1	1	1	1	1	1	1	1	1	0	1	1	1	1	1	1	1	1	1	1
RM27601	180	0	0	0	0	0	0	0	0	1	0	0	0	0	0	0	0	0	0	1	0
RM27601	160	1	1	1	1	1	1	1	1	0	1	1	1	1	1	0	1	1	1	0	1
RM27601	150	0	0	0	0	0	0	0	0	0	0	0	0	0	0	1	0	0	0	0	0
R1M7	185	0	0	1	0	0	1	0	0	0	0	0	0	0	0	0	1	0	0	0	0
R1M7	150	1	1	0	1	1	0	1	1	1	1	1	1	1	1	1	0	1	1	1	1
R1M30	244	1	1	0	1	1	1	1	1	1	1	0	0	0	1	1	1	1	1	1	1
R1M30	197	0	0	1	0	0	0	0	0	0	0	0	0	0	0	0	0	0	0	0	0
R1M30	Null	0	0	0	0	0	0	0	0	0	0	1	1	1	0	0	0	0	0	0	0

Supplementary Table S10: Jaccard's dissimilarity matrix of the twenty indigenous *Joha* rice cultivars based on 66 SSR markers

Cultivar	*Joha (Bihpuria)*	*Kalijeera*	*Ronga Joha*	*Joha (Golaghat)*	*Manimuni Joha*	*Kon Joha (Moran)*	*Keteki Joha*	*Kon Joha-1*	*Soru Joha (Tinsukia)*	*Kon Joha-2*	*Jeera Joha*	*Kon Joha-3*	*Kon Joha-4*	*Kunkuni Joha*	*Kon Joha-5*	*Local Joha*	*Harinarayan*	*Kon Joha (Teok)*	*Kola Joha*
Joha (Bihpuria)																			
Kalijeera	0.49																		
Ronga Joha	0.47	0.49																	
Joha (Golaghat)	0.55	0.54	0.57																
Manimuni Joha	0.47	0.39	0.46	0.44															
Kon Joha (Moran)	0.56	0.4	0.51	0.51	0.4														
Keteki Joha	0.51	0.59	0.66	0.53	0.58	0.57													
Kon Joha-1	0.4	0.35	0.48	0.54	0.24	0.42	0.59												
Soru Joha (Tinsukia)	0.43	0.47	0.38	0.51	0.38	0.52	0.6	0.38											
Kon Joha-2	0.68	0.57	0.7	0.63	0.59	0.62	0.69	0.54	0.62										
Jeera Joha	0.65	0.72	0.73	0.61	0.68	0.75	0.66	0.71	0.71	0.65									
Kon Joha-3	0.51	0.65	0.67	0.65	0.6	0.65	0.59	0.56	0.63	0.68	0.52								
Kon Joha-4	0.54	0.59	0.67	0.65	0.62	0.64	0.61	0.59	0.65	0.7	0.54	0.54							
Kunkuni Joha	0.44	0.39	0.54	0.48	0.35	0.51	0.52	0.35	0.51	0.62	0.64	0.56	0.54						
Kon Joha-5	0.67	0.67	0.72	0.78	0.71	0.75	0.75	0.66	0.69	0.65	0.77	0.72	0.71	0.64					
Local Joha	0.63	0.53	0.56	0.62	0.49	0.57	0.56	0.51	0.58	0.59	0.73	0.71	0.65	0.42	0.71				
Harinarayan	0.47	0.46	0.44	0.46	0.44	0.52	0.56	0.46	0.49	0.66	0.72	0.56	0.6	0.39	0.6	0.53			
Kon Joha (Teok)	0.49	0.44	0.46	0.56	0.37	0.57	0.61	0.4	0.36	0.56	0.76	0.65	0.69	0.4	0.68	0.48	0.4		
Kola Joha	0.51	0.44	0.48	0.6	0.39	0.55	0.58	0.39	0.36	0.63	0.71	0.62	0.63	0.44	0.63	0.53	0.46	0.42	
Kon Joha (Bongaigaon)	0.51	0.44	0.44	0.54	0.39	0.55	0.59	0.33	0.36	0.53	0.72	0.62	0.6	0.42	0.64	0.59	0.44	0.37	0.39
